# *Actinidia arguta* (Sieb. et Zucc.) Planch. ex Miq.: A Review of Phytochemistry and Pharmacology

**DOI:** 10.3390/molecules28237820

**Published:** 2023-11-28

**Authors:** Haifeng Zhang, Kun Teng, Hao Zang

**Affiliations:** 1 School of TCM and Pharmacology Health and Early Childhood Care, Ningbo College of Health Sciences, Ningbo 315100, China; 13429298867@163.com; 2 Green Medicinal Chemistry Laboratory, School of Pharmacy and Medicine, Tonghua Normal University, Tonghua 134002, China

**Keywords:** *Actinidia arguta* (Sieb. et Zucc.) Planch. ex Miq., secondary metabolites, flavonoids, phenolics, pharmacological effects

## Abstract

*Actinidia arguta* (Siebold & Zucc.) Planch ex Miq. (*A. arguta*) is a highly valued vine plant belonging to the *Actinidia* lindl genus. It is extensively utilized for its edible and medicinal properties. The various parts of *A. arguta* serve diverse purposes. The fruit is rich in vitamins, amino acids, and vitamin C, making it a nutritious and flavorful raw material for producing jam, canned food, and wine. The flowers yield volatile oils suitable for essential oil extraction. The leaves contain phenolic compounds and can be used for tea production. Additionally, the roots, stems, and leaves of *A. arguta* possess significant medicinal value, as they contain a wide array of active ingredients that exert multiple pharmacological and therapeutic effects. These effects include quenching thirst, relieving heat, stopping bleeding, promoting blood circulation, reducing swelling, dispelling wind, and alleviating dampness. Comprehensive information on *A. arguta* was collected from scientific databases covering the period from 1970 to 2023. The databases used for this review included Web of Science, PubMed, ProQuest, and CNKI. The objective of this review was to provide a detailed explanation of *A. arguta* from multiple perspectives, such as phytochemistry and pharmacological effects. By doing so, it aimed to establish a solid foundation and propose new research ideas for further exploration of the plant’s potential applications and industrial development. To date, a total of 539 compounds have been isolated and identified from *A. arguta*. These compounds include terpenoids, flavonoids, phenolics, phenylpropanoids, lignin, organic acids, volatile components, alkanes, coumarins, anthraquinones, alkaloids, polysaccharides, and inorganic elements. Flavonoids, phenolics, alkaloids, and polysaccharides are the key bioactive constituents of *A. arguta*. Moreover, phenolics and flavonoids in *A. arguta* exhibit remarkable antioxidant, anti-inflammatory, and anti-tumor properties. Additionally, they show promising potential in improving glucose metabolism, combating aging, reducing fatigue, and regulating the immune system. While some fundamental studies on *A. arguta* have been conducted, further research is necessary to enhance our understanding of its mechanism of action, quality evaluation, and compatibility mechanisms. A more comprehensive investigation is highly warranted to explore the mechanism of action and expand the range of drug resources associated with *A. arguta*. This will contribute to the current hot topics of anti-aging and anti-tumor drug research and development, thereby promoting its further development and utilization.

## 1. Introduction

**Figure 1:**
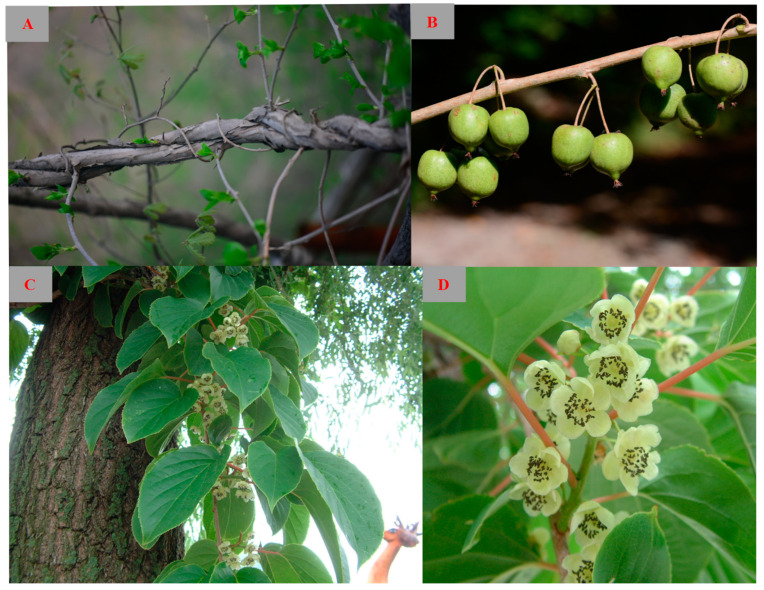
Stems (**A**), fruit (**B**), leaves (**C**), and flowers (**D**) of *Actinidia arguta*.

The *Actinidia* lindl genus belongs to the Actinidiaceae family and comprises more than 54 species, including deciduous, semi-deciduous, and evergreen vines. *Actinidia arguta* (Sieb. et Zucc.) Planch. ex Miq. (*A. arguta*), which is commonly known as kiwiberry (Figure 1), is a large deciduous vine found in China, Korea, Japan, and Russia. It thrives in mixed forests and well-watered environments, particularly at altitudes ranging from 500 to 1500 m [1]. Another noteworthy plant within this genus is *Actinidia chinensis* Planch., which contributes to China’s role as the world’s leading kiwifruit producer [2]. These two species display distinct morphological characteristics, making them easily distinguishable. The primary difference lies in the size and appearance of the fruit. *A. arguta* produces relatively smaller, smooth, green-skinned fruit, while *Actinidia chinensis* bears larger, brown-skinned fruit covered in fuzz [3].

The roots of *A. arguta* possess significant medicinal properties [4]. Its benefits have been documented in the “Dietary Materia Medica” of the Tang Dynasty and the “Compendium of Materia Medica” of the Ming Dynasty. Traditionally, the roots have been used to quench thirst, relieve heat, stop bleeding, promote blood circulation, reduce swelling, dispel wind, and alleviate dampness. In modern clinical practice, it is employed in the treatment of ailments such as rheumatism, lymphoid tuberculosis, esophageal cancer, gastric cancer, and breast cancer. Additionally, *A. arguta* is valued both as an ornamental tree species and a fruit tree. Its leaves contain polyphenolic compounds suitable for tea preparation [5]. The fruit, which is known for its potent antipyretic and astringent effects, is also highly nutritious and widely consumed [6]. Its small size and seeds make it ideal for fresh consumption, as well as for making jam, canned food, and wine [7,8,9]. The flesh is tender and juicy, offering a delightful sweet and sour flavor. Abundant in amino acids, vitamins, and minerals, particularly vitamin C, which surpasses other fruits by severalfold, the *A. arguta* fruit is a valuable ingredient for the development of functional health foods [10,11].

Extensive research identified and isolated over 500 compounds from *A. arguta*, spanning various categories, such as terpenoids, phenolics, flavonoids, phenylpropanoids, lignin, organic acids, volatile oils, steroids, anthraquinones, coumarins, alkaloids, and amino acids [12]. Notably, terpenoids, phenolics, and flavonoids have garnered significant attention due to their immense potential for development and utilization.

While studies summarizing the phytochemistry and pharmacological effects of *A. arguta* exist, certain information gaps and inadequacies need to be addressed. These include an incomplete listing of chemical components and insufficient details regarding their chemical structures. Additionally, the description of the pharmacological mechanism of *A. arguta* lacks thoroughness. A previous report discussed the chemical components and pharmacological effects of *A. arguta* [13]. However, this previous review only provided brief introductions to the names of over 60 chemical components, their total extracts, and a concise overview of the anti-tumor, antioxidant, and hypoglycemic effects. In contrast, our review encompasses a total of 539 components, complete with structural information for each compound. Furthermore, our review delves into distinct classifications of pharmacological research, providing an up-to-date and comprehensive observational perspective on *A. arguta*.

Therefore, we conducted a comprehensive literature review to address the aforementioned gaps by offering a comprehensive examination of the phytochemistry and pharmacological effects of *A. arguta*. We aimed to inspire future research on *A. arguta* while providing valuable references for the rational utilization of its resources and the efficient development of related products.

## 2. Materials and Methods

To ensure the reliability and integrity of the information gathered for this review, we meticulously collected data from numerous databases including Web of Science, PubMed, ProQuest, and the China National Knowledge Infrastructure (CNKI). Our literature search encompassed articles published in peer-reviewed journals, Ph.D. dissertations, master’s theses, conference papers, and classic texts of Chinese herbal medicines. To maximize the breadth of our research, we employed specific keywords during the literature search, such as *Actinidia arguta*, phytochemistry, secondary metabolites, pharmacology, biological activity, safety, toxicology, medicinal uses, and other related terms. This enabled us to retrieve a comprehensive range of relevant studies published between 1970 and November 2023.

## 3. Phytochemistry

Given the successful isolation and identification of numerous bioactive compounds from the roots, stems, leaves, and fruit of *A. arguta*, there is a growing interest in utilizing the fruit of *A. arguta* as raw materials for health food and the roots, stems, and leaves of *A. arguta* as medicinal resources [2,3,4,5,6,7,8,9,10,11]. *A. arguta* contains a diverse range of compounds, with over 500 compounds isolated from the plant according to literature reports [12]. These compounds can be broadly categorized into eight types, including terpenoids, flavonoids, phenolics, phenylpropanoid and lignin compounds, organic acids, and volatile compounds. This emphasizes the abundant potential of *A. arguta* as a source of bioactive ingredients that can be further explored in drug development, functional food production, and nutritional applications [13].

### 3.1. Terpenoids

**Figure 2:**
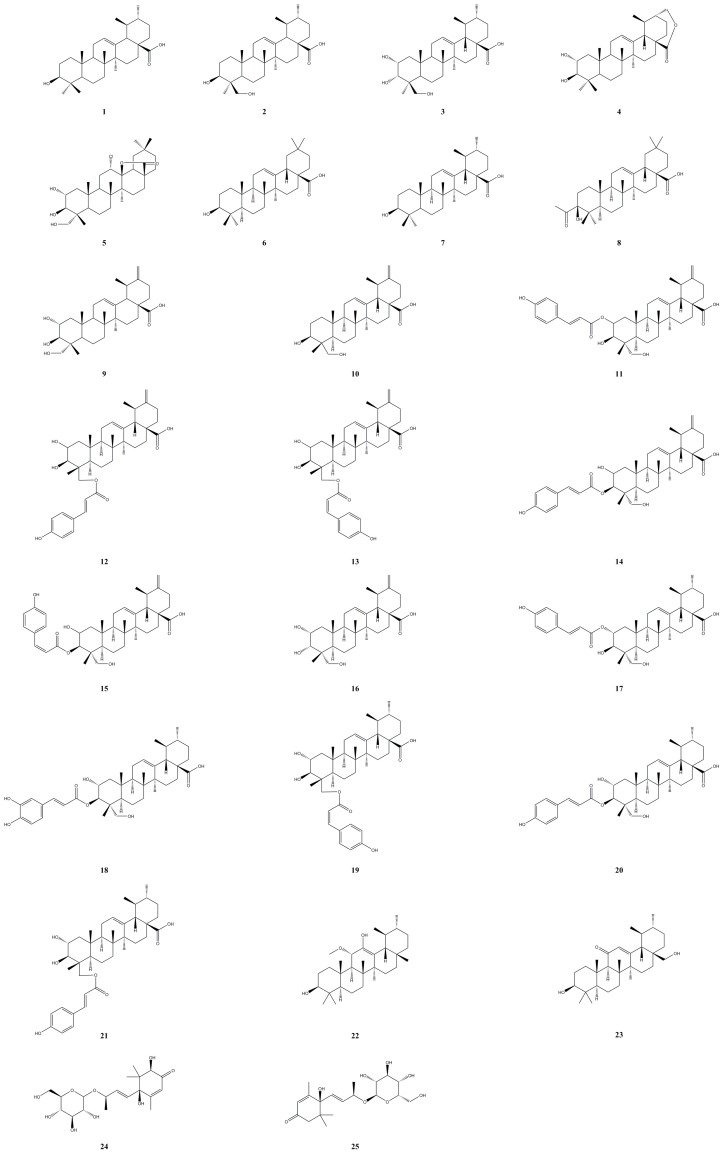
Chemical structures of terpenoids isolated from *Actinidia arguta*. Chemical structures were drawn using Chemdraw Professional 15.0 software.

Terpenoids are primarily found in *A. arguta*, consisting mainly of triterpenoids and their glycosides, with a small amount of sesquiterpenoids. These terpenoids are primarily isolated from the roots and leaves of the plant. With 25 identified terpenoids (Table 1, Figure 2), primarily belonging to the ursane and oleanane types, ursolic acid and oleanolic acid were initially isolated from the leaves [14] and roots [15] of *A. arguta*. Shi et al. (1993) isolated and identified three triterpenoids from the leaves of *A. arguta*: 3*β*-hydroxyurs-12-en-28-oic acid (**1**), 3*β*,24-dihydroxyurs-12-en-28-oic acid (**2**) [16], and 2*α*,3*α*,24-trihydroxyurs-12-en-28-oic acid (**3**) [17]. Teng et al. (2019) employed high-performance liquid chromatography-mass spectrometry (HPLC-MS) analysis to identify two triterpenoid compounds, namely, 2*α*,3*β*-dihydroxyurs-12-en-28,30-olide (**4**) and 12*α*-chloro-2*α*,3*β*,23-tetrahydroxyolean-28-oic acid-13-lactone (**5**) [18]. Zhao et al. (1994) isolated and identified acetyl oleanolic acid from the stem of *A. arguta* [19]. Ahn et al. (2020) isolated eight known triterpenoids and seven new triterpenoids, including actiniargupenes A−F (**10**–**12**, **17**–**19**) and dehydroisoactinidic acid (**16**), from the leaves of *A. arguta*. All the compounds (100 μM) demonstrated inhibitory effects on *α*-glucosidase activity. Among them, 3-O-*trans*-*p*-coumaroylasiatic acid (**20**) outperformed acarbose, while actiniargupene E (**18**) and actiniargupene A (**10**) exhibited comparable effects to acarbose [20]. Li et al. (2020) first isolated a new norsesquiterpene glycoside, namely, (2*R*,6*R*,9*R*)-trihydroxy-megastigmane-4,7*E*-dien-3-one-9-O-*β*-D-gluco-pyranoside (**24**), and a monoterpenoid, namely, (6*S*, 9*R*)-roseoside (**25**), from the fruit of *A. arguta* [21].

### 3.2. Flavonoids

**Figure 3:**
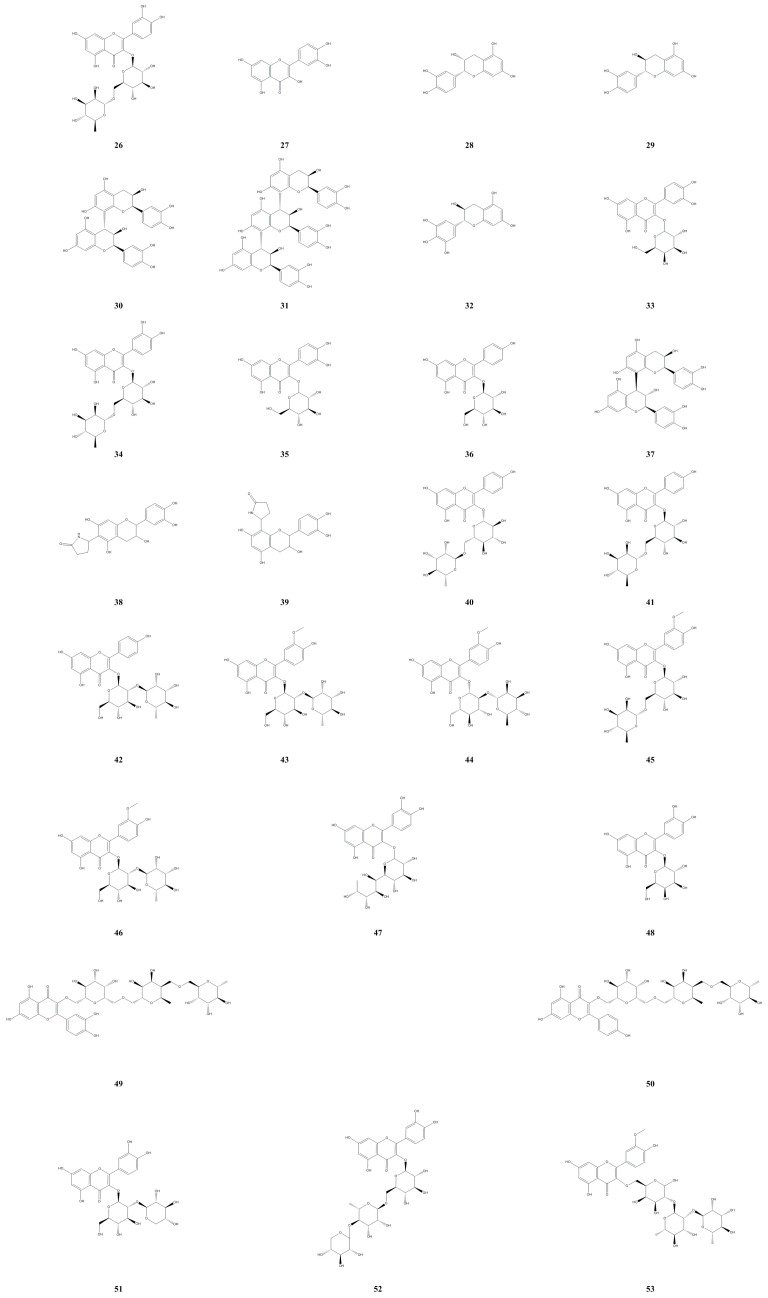
Chemical structures of flavonoids isolated from *Actinidia arguta*. Chemical structures were drawn using Chemdraw Professional 15.0 software.

In recent years, 28 flavonoids were isolated and identified from the roots, fruit, and leaves of *A. arguta* (Table 2, Figure 3). One study reported the identification of rutin (**26**) and quercetin (**27**) using HPLC technology and comparing them with reference materials [22]. HPLC-MS analysis was also utilized to identify 15 flavonoids from *A. arguta*, including kaempferol-3-O-rutinoside (+) (**40**), kaempferol-3-O-rutinoside (−) (**41**), kaempferol-3-O-neohesperidoside (**42**), isorhamnetin-3-O-neohesperidoside (+) (**43**), isorhamnetin-3-O-neohesperidoside (−) (**44**), isorhamnetin-3-O-rutinoside (**45**), isorhamnetin-3-O-neohesperidoside (**46**), quercetin-3-O-rhamnoglucoside (**47**), and isorhamnetin-3-O-*α*-L-rhamnopyranosyl-(1-3)-*α*-L-rhamnopyranosyl-(1-6)-*β*-D-galactopyranoside (**53**) [23]. Other isolated flavonoids include proanthocyanidin B2 (**30**), proanthocyanidin C1 (**31**), (+)-gallocatechin (**32**), quercetin-3-O-galactoside (**33**), quercetin-3-O-rutinoside (**34**), and quercetin-3-O-glucoside (**35**); the presence of these compounds is the reason for strong antioxidant activity of *A. arguta*, as evidenced by peroxyl radical scavenging capacity and cellular antioxidant activity assays [24]. Additionally, Li et al. (2020) isolated two flavonoid monosaccharide glycosides from the fruit: quercetin-3-O-*β*-D-galactopyranoside (**48**) and astragalin (**36**) [21]. From the roots of *A. arguta*, five flavonoids were isolated and identified: (−)-*epi*-catechin (**28**), (+)-catechin (**29**), procyanidin B4 (**37**), 6-(2-pyrrolidinone-5-yl)-(−)-epicatechin (**38**), and 8-(2-pyrrolidinone-5-yl)-(−)-epicatechin (**39**). Among them, **37** and **29** exhibited the most potent inhibitory activity against advanced glycation end product formation, with half-maximal inhibitory concentration (IC_50_) values of 10.1 μM and 13.6 μM, respectively. Flavonoids **38**, **39**, and **28** also demonstrated significant activities in the assay, with IC_50_ values of 36.0, 47.8, and 125.2 μM, respectively, suggesting their potential in the treatment of diabetes-related complications and diseases [25]. From the leaves of *A. arguta*, four flavonoids were sequentially isolated and identified: quercetin-3-O-[*α*-rhamnopyranosyl-(1-4)-rhamnopyranosyl-(1-6)-*β*-galactopyranoside (**49**), kaempferol-3-O-[*α*-rhamnopyranosyl-(1-4)-rhamnopyranosyl-(1-6)-*β*-galactopyranoside (**50**), quercetin 3-sambubioside (**51**), and quercetin 3-O-*β*-D-[2-O-*β*-D-xylopyranosy-6-O-*α*-L-rhamnopyranosyl] glucopyranoside (**52**) [26,27].

### 3.3. Phenolic Compounds

**Figure 4:**
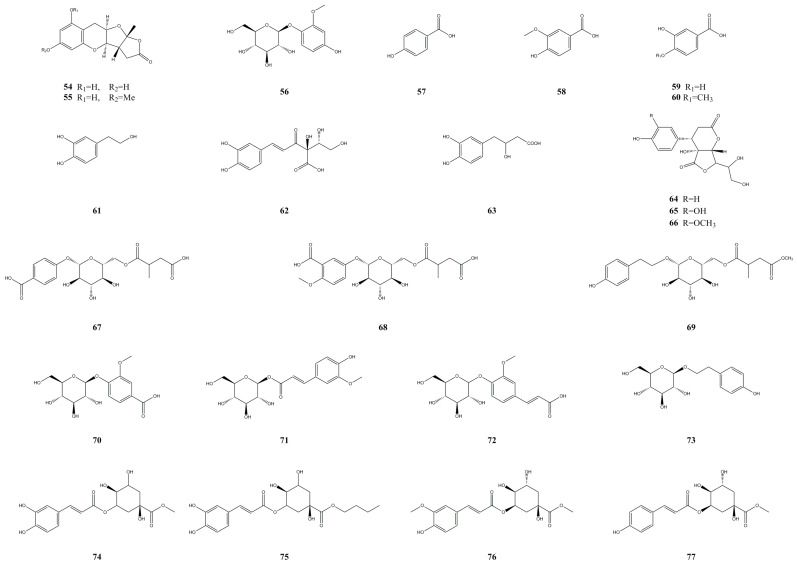
Chemical structures of phenolic compounds isolated from *Actinidia arguta*. Chemical structures were drawn using Chemdraw Professional 15.0 software.

Phenolic compounds are widely occurring secondary metabolites in plants and hold significant pharmacological and nutritional importance [28]. So far, researchers have isolated and identified 24 phenolic compounds from the roots, fruit, and leaves of *A. arguta* (Table 3, Figure 4). Through the use of HPLC-MS technology, three phenolic compounds were identified in the roots: planchols A and B (**54** and **55**) and isotachioside (**56**) [18]. From the leaves of *A. arguta*, researchers isolated 11 phenolic compounds, namely, *p*-hydroxybenzoic acid (**57**); vanillic acid (**58**); protocatechuic acid (**59**); isovanillic acid (**60**); hydroxytyrosol (**61**) [29]; caffeoylthreonic acid (**62**); salvianic acid A (**63**) [30]; maysedilactones A, B, and D (**64**–**66**) [31]; and rhodioloside (**73**) [21]. Additionally, 10 phenolic compounds were identified in the fruit, including argutinosides J–L (**67**–**69**) [32], vanillic acid-4-O-*β*-D-glucopyranoside (**70**), 1-O-feruloyl-*β*-D-glucopyranoside (**71**), ferulic acid-4-O-*β*-D-glucopyranoside (**72**), 5-O-caffeoyl quinic acid methyl ester (**74**), 5-O-caffeoyl quinic acid butyl ester (**75**), 5-O-feruloyl quinic acid methyl ester (**76**), and 5-O-coumaroyl quinic acid methyl ester (**77**) [21]. Phenols **57**–**59** (100 μM) exhibited weak 2,2-diphenyl-1-picrylhydrazyl (DPPH) radical scavenging and *α*-glucosidase inhibitory activities, while **60** and **61** demonstrated strong DPPH radical scavenging and *α*-glucosidase inhibitory activities [29]. Phenols **64**–**66** displayed *α*-glucosidase inhibitory and DPPH radical scavenging activities. Specifically, **66** inhibited *α*-glucosidase activity by 43% and exhibited an antioxidant effect of up to 89% at a concentration of 100 μM. This study suggests that **64**–**66** may be effective in treating oxidative stress related to metabolic diseases [31]. On the other hand, **67** and **68** showed mild DPPH radical scavenging activities, with percentages of 52.1% and 68.2% respectively, at a concentration of 500 μM, while **69** exhibited weak DPPH activity [32].

### 3.4. Phenylpropanoid and Lignin Compounds

**Figure 5:**
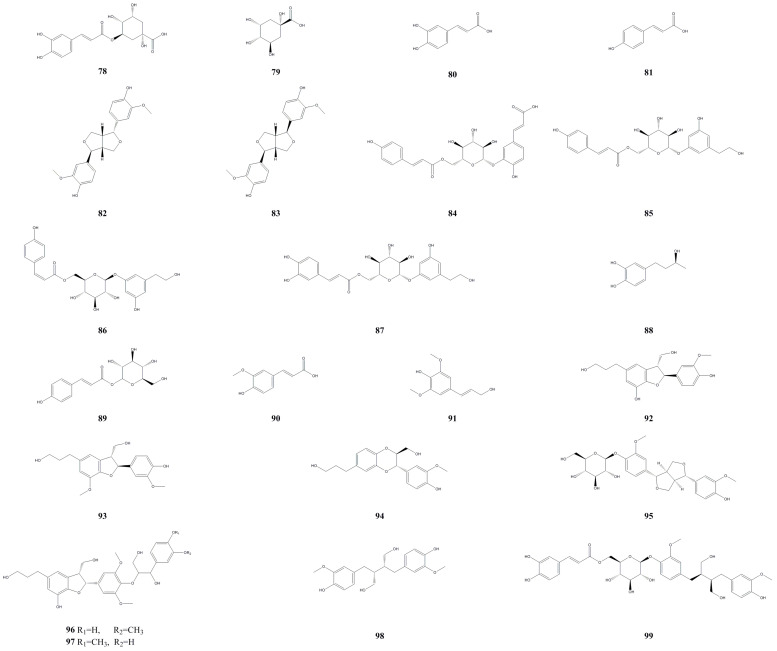
Chemical structures of phenylpropanoid and lignin compounds isolated from *Actinidia arguta*. Chemical structures were drawn using Chemdraw Professional 15.0 software.

Phenylpropanoid and lignin compounds primarily function in regulating plant growth and resistance against viruses [33]. For the first time, researchers isolated and identified 10 phenylpropanoid compounds from the leaves of *A. arguta* (Table 4, Figure 5). These compounds include argutosides A–D (**84**–**87**), (−)-rhodolatouchol (**88**), *p*-*E*-coumaric acid-9-O-glucopyranoside (**89**), *E*-ferulic acid (**90**), 3,5-dimethoxy-4-hydroxycinnamic alcohol (**91**), caffeic acid (**80**), and *trans*-4-hydroxycinnamic acid (**81**) [29], and chlorogenic acid (**78**) [34]. Furthermore, quinic acid (**79**) was identified using HPLC-MS analysis of the roots and fruit [35]. From the leaves, researchers also isolated and identified 10 lignin compounds, namely, pinoresinol (**83**), 7*S*,8*R*-cedrusin (**92**), dehydroconiferyl alcohol (**93**), (7*S*,8*S*)-3-methoxy-3′,7-epoxy-8,4′-oxyneoligna-4,9,9′-triol (**94**), pinoresinol 4-O-*β*-glucopyranoside (**95**), alutaceuol (**96**), alutaceuol isomer (**97**), (−)-(2*R*,3*R*)-secoisolariciresinol (**98**), glehlinoside F (**99**), and epipinoresinol (**83**) [25,29]. When the concentration of chlorogenic acid in the leaves of *A. arguta* ranges from 0.2 to 1.0 mg/mL, its ability to scavenge DPPH gradually increases, reaching a maximum scavenging rate of 92.0%. At lower concentrations, chlorogenic acid exhibits a strong ability to scavenge hydroxyl radicals, with rates exceeding 80%. As the concentration increases, the effect of increasing the scavenging rate of hydroxyl radicals becomes less significant, and the maximum scavenging rate reaches 95.0% [34].

### 3.5. Organic Acids (Esters)

**Figure 6:**
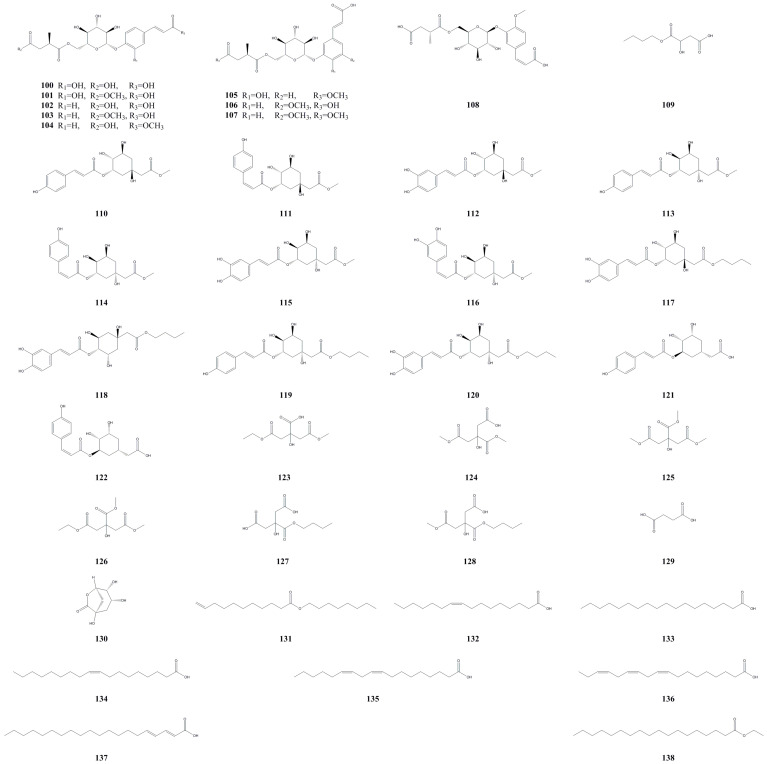
Chemical structures of organic acids (esters) isolated from *Actinidia arguta*. Chemical structures were drawn using Chemdraw Professional 15.0 software.

In a separate study, 29 organic acid compounds were isolated and identified for the first time from the fruit of *A. arguta* (Table 5, Figure 6). Among them, there are 10 succinic acid derivatives (**100**–**109**), 11 quinic acid derivatives (**110**–**120**), 2 shikimic acid derivatives (**121**–**122**), and 6 citric acid derivatives (**123**–**128**). The compounds were evaluated for their NF-*κ*B transcriptional inhibitory activities using lipopolysaccharide (LPS)-induced RAW 264.7 macrophages. Among the four groups of organic acid derivatives, quinic acid derivatives with phenylpropanoids exhibited the most potent NF-*κ*B inhibitory activities, with an IC_50_ value of 4.0 μM, while the others showed weak activities. Two isolated compounds, namely, **111** and **115**, demonstrated NF-*κ*B inhibitory activities, with IC_50_ values of 8.7 and 4.9 μM, respectively [36]. Additionally, ethyl stearate (**138**) was isolated and identified from the fruit by Park et al. (2011) [37]. Furthermore, researchers identified *γ*-quinide (**130**) [18], octeyl-10-undecylenate (**131**) [38], and succinic acid (**129**) [14] from the roots, stems, and leaves. There have also been reports of identifying six fatty acids from the sprouts, namely, palmitoleic acid (**132**), stearic acid (**133**), oleic acid (**134**), *α*-linoleic acid (**135**), *α*-linolenic acid (**136**), and eicosadienoic acid (**137**) [39]. Moreover, compounds **135**, **136**, and **138** exhibit downregulatory effects on IL-4 production in A23187-stimulated RBL-2H3 cells without inducing cytotoxicity. *α*-linolenic acid shows the highest downregulatory effect. Both **135** and **136** are present as glycerol esters in animal and plant oils, as well as in dark green plants. They are essential fatty acids necessary for the human body’s nutritional requirements. The intake of these two compounds in different proportions can impact adult blood sugar levels [40].

### 3.6. Volatile Compounds

**Figure 7:**
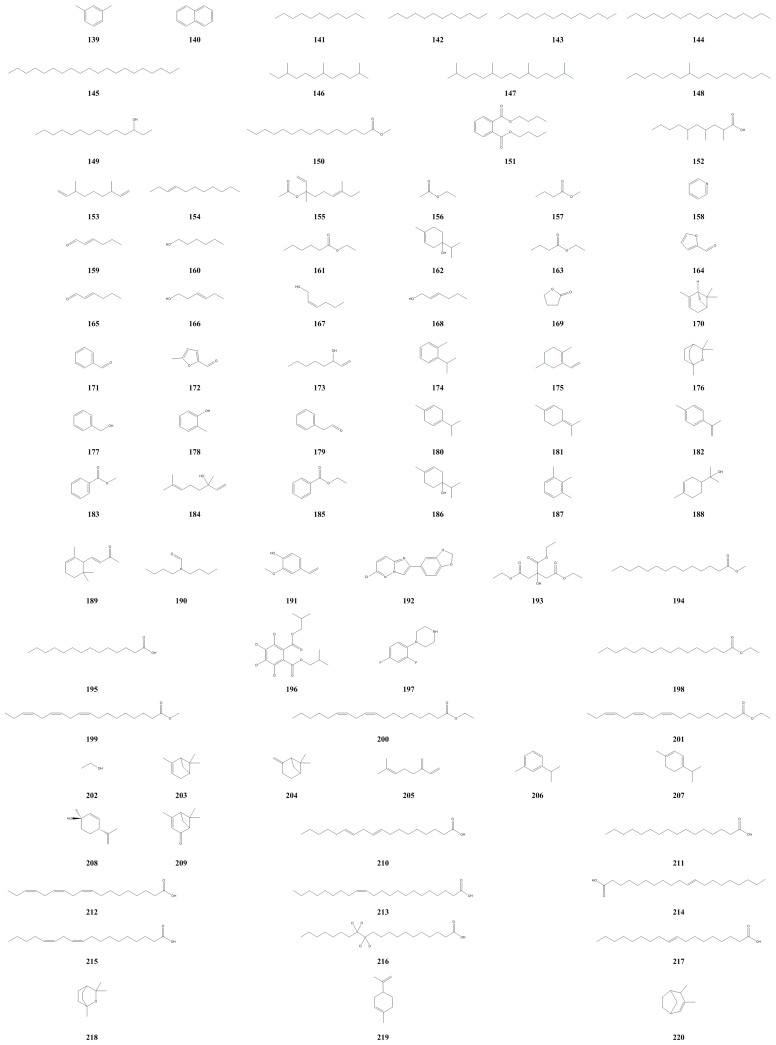

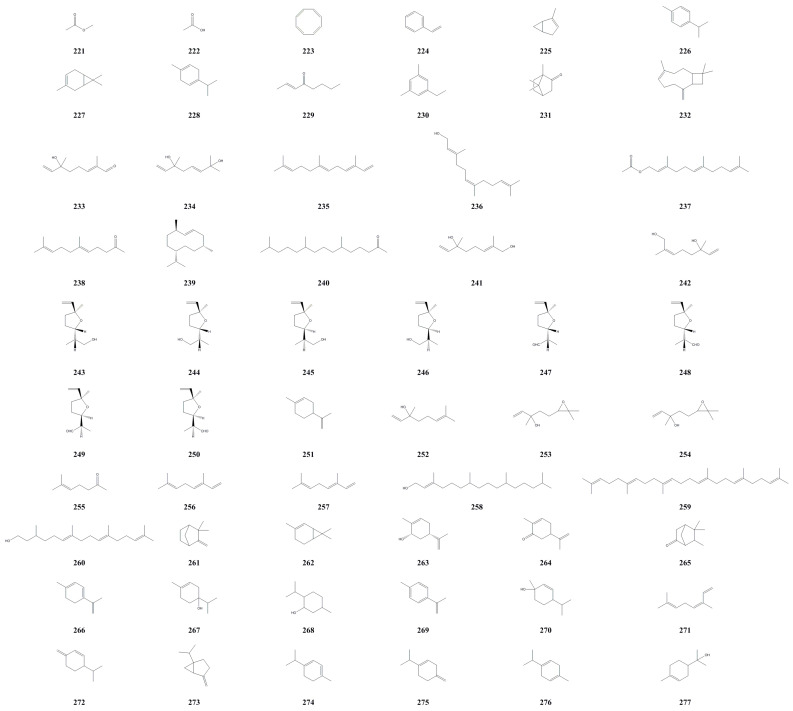

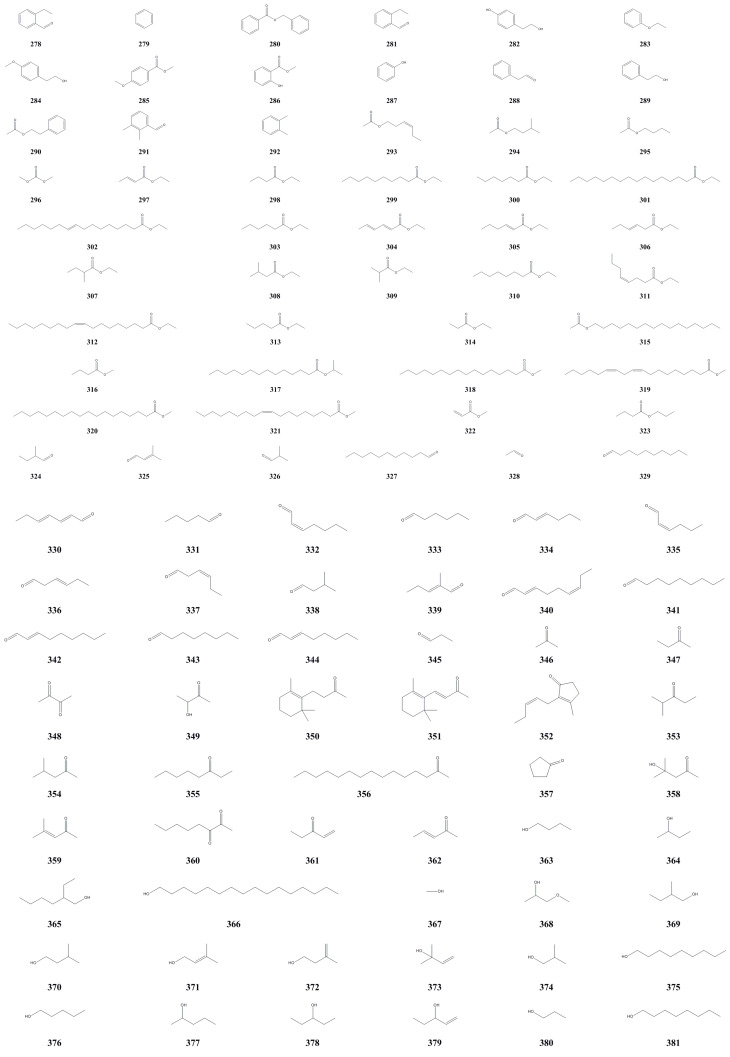

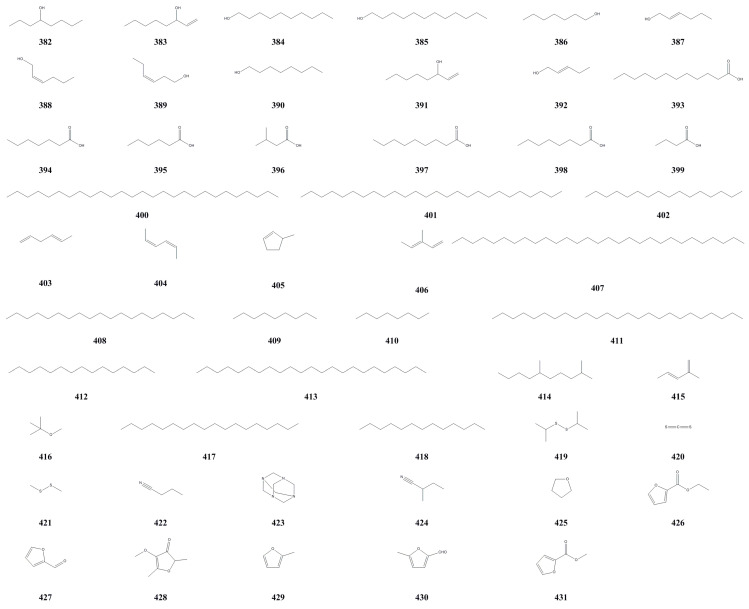

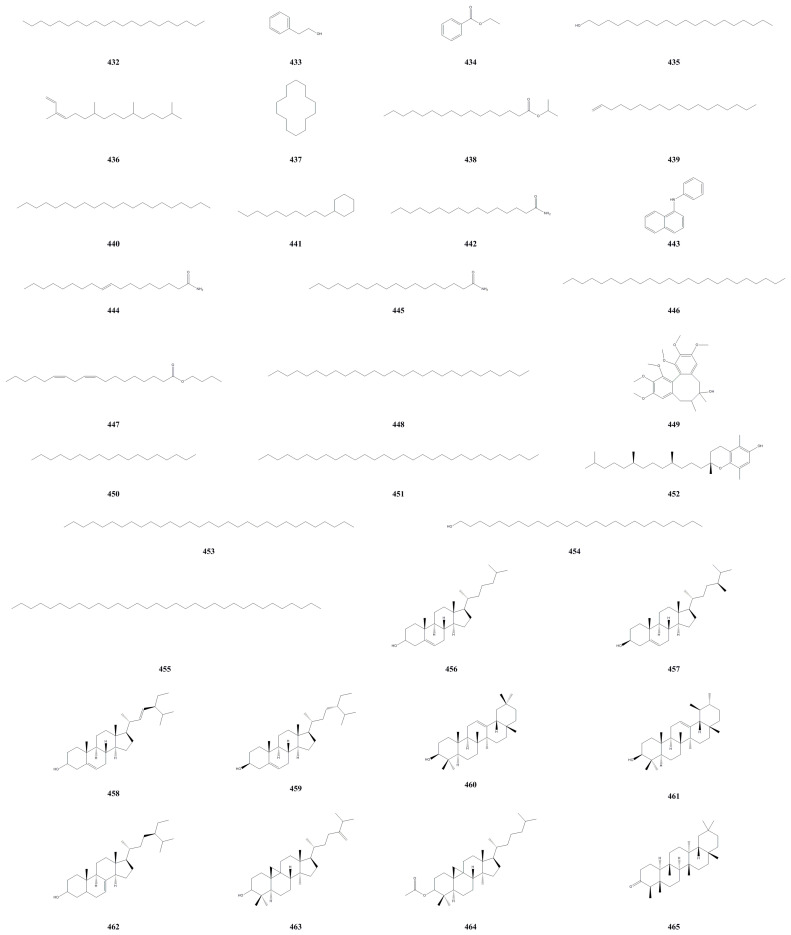
Chemical structures of volatile components isolated from *Actinidia arguta*. Chemical structures were drawn using Chemdraw Professional 15.0 software.

All volatile compounds, except for *n*-docosane, were identified using gas chromatography-mass spectrometry (GC-MS) analysis. So far, 327 volatile compounds have been analyzed and identified from *A. arguta* (Table 6, Figure 7), primarily from fruit, with a smaller portion from roots and seeds. Matich et al. (2003) identified over 200 volatile components from flowers and fruit, mainly including linalool derivatives and sesquiterpenoids [41]. Yang et al. (2012) also identified 32 volatile components from the fruit, mainly phenolics, alcohols, and alkenes [42]. Other researchers isolated 12 components from the fruit, predominantly lipids and alcohols. Notably, ethyl butyrate accounts for a significant relative content of 86.89%, which gives the fruit its strong aroma. Although ethyl butyrate is widely used in the food, spice, and tobacco industries, the synthetic form raises concerns about its toxicity. Given the current preference for natural spices, the high relative content of ethyl butyrate in *A. arguta* volatile oil presents an excellent opportunity for natural extraction [43]. Xin et al. (2009) utilized a solid-phase microextraction device and employed GC-MS to identify 21 volatile components in *A*. *arguta* [44]. Sun et al. (2012) isolated 10 volatile components from the fruit [45]. Recently, Wang et al. (2022) discovered 33 volatile components from the fruit [46]. Additionally, 13 volatile components were extracted from the fruit and seeds of *A. arguta* [47,48,49]. Yang et al. (2000) identified 17 compounds from the roots, mainly consisting of aliphatic compounds [50].

### 3.7. Other Compounds

**Figure 8:**
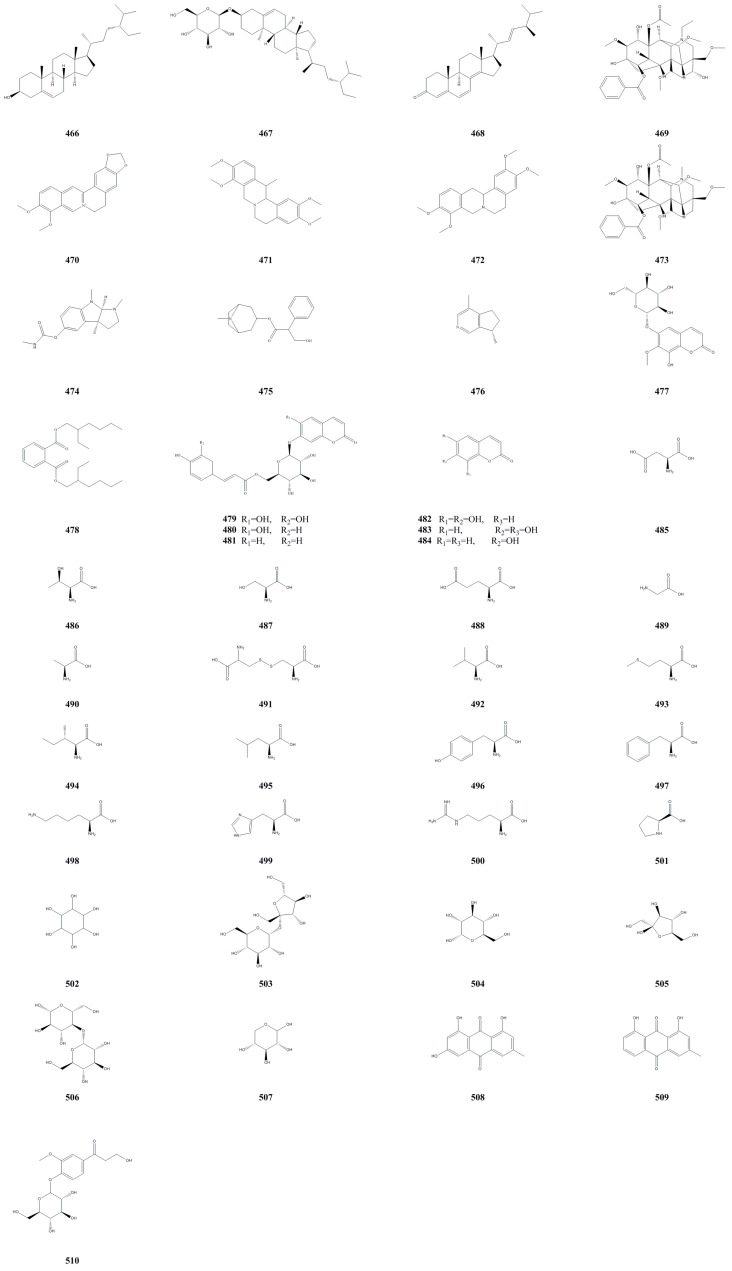
Chemical structures of other compounds isolated from *Actinidia arguta*. Chemical structures were drawn using Chemdraw Professional 15.0 software.

Apart from the aforementioned compound types, an additional 45 different types of compounds were also isolated from *A. arguta* (Table 7, Figure 8). Most of these compounds were obtained from the fruit, while a few were derived from its roots, stems, leaves, buds, and seeds. These compounds primarily include alkaloids [51], anthraquinones [52], coumarins [18,29], amino acids [53], sterols [17,19], sugars, and glycols [39,54]. Notably, argutosides E (**479**), esculetin (**482**), and 7,8-dihydroxycoumarin (**483**) exhibited potent antioxidant and *α*-glucosidase inhibitory activities. However, eculetin 7-O-(6′-O-*trans*-coumaroyl)-*β*-glucopyranoside (**480**), umbelliferone 7-O-(6′-O-*trans*-coumaroyl)-*β*-glucopyranoside (**481**), 7,8-dihydroxycoumarin (**483**), and umbelliferone (**484**) displayed moderate antioxidant and *α*-glucosidase inhibitory activity.

### 3.8. Inorganic Elements

The report indicates that *A. arguta* contains 29 inorganic elements (Table 8), many of which have beneficial effects on the human body [55].

## 4. Pharmacological Activities

As both a medicinal and edible plant, *A. arguta* not only bears edible fruit but its entire plant can also be employed for medicinal purposes. Modern pharmacological studies verified various pharmacological effects of *A. arguta*, including antioxidant, anti-inflammatory, anti-tumor, anti-aging, anti-fatigue, hypoglycemic, lipid-lowering, antibacterial, anti-glycation, anti-radiation, and immune regulation activities.

### 4.1. Antioxidant Activity

The continuous generation of reactive oxygen species (ROS) during oxidative metabolism is regarded as a major contributor to human aging [56]. Excessive accumulation of ROS in organisms may lead to oxidative stress, causing immune injury, rheumatoid arthritis, and atherosclerosis [57]. However, there is growing evidence suggesting that synthetic antioxidants can result in liver damage and even cancer [58]. Consequently, the search for natural antioxidants from plants has gained prominence in recent years [59,60,61,62].

An et al. (2016) extracted total flavonoids and polyphenols from three varieties of *A. arguta* and assessed their antioxidant capacities using DPPH, 2,2′-azino-bis(3-ethylbenzothiazoline-6-sulfonic acid) diammonium salt (ABTS), and oxygen radical absorbance capacity (ORAC) methods. The results indicated that every 100 g of fresh fruit possessed an antioxidant capacity equivalent to containing 203.4 mg, 135.5 mg, and 115.0 mg of vitamin C, respectively [63]. Additionally, the addition of citric acid was observed to enhance the extraction rate of total polyphenols from *A. arguta*, thereby augmenting its antioxidant activity [64]. The flavonoids of *A. arguta* effectively scavenge DPPH at relatively low concentrations, with the EC_50_ value reached at a concentration of 174.2 mg/L, which is similar to vitamin C. Additionally, at a concentration of 750 mg/L, the scavenging rate of DPPH exceeds 90%. Furthermore, *A. arguta* demonstrates a certain ability to scavenge hydroxyl radicals and superoxide anion free radicals, which increases with the concentration of flavonoids [65].

A separate study extracted quercetin from *A. arguta* and evaluated its antioxidant capacity. The results show no significant difference in the total antioxidant capacity between quercetin and vitamin C at the same molar concentration in vitro. However, the IC_50_ of quercetin and vitamin C in the anti-lipid peroxidation assay was 0.79 mg/mL and 1.41 mg/mL, respectively, indicating the former’s superior strength. Moreover, when combined, quercetin and vitamin C exhibited synergistic antioxidant effects. Quercetin’s antioxidant capacity was further evaluated in vivo using a carbon tetrachloride-induced mouse oxidative liver injury model experiment. The results demonstrated that quercetin had significantly better antioxidant capacity in vivo compared with vitamin C. At the same time, combining quercetin with vitamin C resulted in enhanced antioxidant abilities [66]. This could be attributed to quercetin being a lipophilic antioxidant that is primarily distributed near the biofilm’s surface, while vitamin C, which is a hydrophilic antioxidant, is located outside the membrane where it can scavenge ROS that diffuse outside the membrane [67].

Lee et al. (2014) analyzed the antioxidant activities of different solvent extracts from the stem of *A. arguta*. The ethyl acetate fraction (IC_50_, 14.28 μg/mL) and *n*-butanol fraction (IC_50_, 48.27 μg/mL) exhibited high DPPH scavenging activity. In addition, the ethyl acetate fraction (200 μg/mL) effectively inhibited nitric oxide (NO) production in RAW 264.7 cells induced by LPS, in contrast with other fractions [68]. Gao et al. (2019) measured the antioxidant capacities of different solvent extracts (ethyl acetate, n-butanol, water, methanol, and ethanol) from the adventitious roots of *A. arguta*. The ethyl acetate extract showed the strongest antioxidant capacity, with a DPPH scavenging rate of 88.09% at a concentration of 0.1 mg/mL. The ABTS scavenging rate at a concentration of 1.0 mg/mL was 95.62%. The chelating ability of iron ions was positively correlated with the concentration of the extract [69]. Khromykh et al. (2022) studied the antioxidant activities of components such as polyphenols in the peels and pulps of *A. arguta*. The results indicated that the peels had stronger reducing power and total antioxidant capacity compared with the pulps [70]. The best processing extract of *A. arguta* exhibited exceptional antioxidant and antiradical activities, including ABTS, ferric-reducing antioxidant power (FRAP), superoxide anion radical, hypochlorous acid, and peroxyl radical scavenging [71].

Plant polysaccharides, in addition to multifunctional compounds, such as flavonoids, phenolics, and anthraquinones, also possess significant antioxidant capacity [72]. Polysaccharides from *A. arguta* fruit exhibited a strong scavenging ability for DPPH and alkyl radicals, with IC_50_ values of 0.497 mg/mL and 0.547 mg/mL, respectively. At a concentration of 1 mg/mL, the polysaccharides showed scavenging rates of 86.4% and 87.1% for DPPH and alkyl radicals, similar to vitamin C [73]. Moreover, polysaccharides from *A. arguta* fruit demonstrated the ability to scavenge hydroxyl radicals [74], indicating their strong antioxidant potential and promising prospects for development. Polysaccharides extracted from *A. arguta* leaves and stems also exhibited DPPH scavenging activities, with IC_50_ values of 0.71 mg/mL and 0.72 mg/mL, respectively. These findings suggest that polysaccharides derived from *A. arguta* leaves and stems could be further developed and utilized as natural antioxidants [75].

The alkaloids found in *A. arguta* show scavenging effects on DPPH, hydroxyl radicals, and superoxide anion radicals. These alkaloids were also found to inhibit lipid peroxidation and possess a strong iron ion reduction ability [51]. The volatile components of *A. arguta* demonstrated certain antioxidant activity. The essential oil of *A. arguta* exhibited strong scavenging activity with DPPH (IC_50_ = 117.60 μg/mL), which was comparable with the synthetic antioxidant butylated hydroxytoluene (BHT) [46]. Furthermore, the storage temperature was found to impact the antioxidant activity of *A. arguta*. Storage at 0 °C significantly inhibited the browning and respiratory intensity of *A. arguta* compared with 5 °C and 10 °C. Additionally, storage at 0 °C maintained higher fruit hardness and vitamin C, glutathione (GSH), and flavonoid contents while inhibiting relative conductivity; malondialdehyde (MDA) content; and peroxidase, and polyphenol oxidase activities. Storage at 0 °C also maintained higher superoxide dismutase (SOD), catalase, and glutathione reductase activities [76].

### 4.2. Anti-Inflammatory Activity

Inflammatory reactions can protect the human body from bacteria and tumors. However, chronic inflammation resulting from the continuous activation of macrophages can lead to serious health issues, including heart disease, gastrointestinal problems, and a sore throat. *A. arguta* contains various anti-inflammatory compounds, such as (+)-catechin, chlorogenic acid, (−)-epi-catechin, quercetin, rutin, and caffeic acid. Experimental results suggest that the chloroform layer of *A. arguta* stems exerts anti-inflammatory effects by inhibiting mitogen-activated protein kinase phosphorylation and the nuclear translocation of NF-*κ*B [77]. In vitro experiments showed that the methanol extract of *A. arguta* leaves (12.5, 25, and 50 μg/mL) specifically inhibits NLRP3 ubiquitination, thereby suppressing the secretion of caspase-1 and IL-1*β*. This conclusion was also confirmed in in vivo experiments on a mouse model of peritonitis [78]. The results of anti-inflammatory activity research showed that when the total triterpenoid concentration of *A. arguta* branches reached 4 mg/mL, the inhibition rates of hyaluronidase activity and bovine serum albumin denaturation reached 81.48% and 71.09%, respectively. The anti-inflammatory activity was slightly lower than that of the positive control, namely, diclofenac sodium. The results confirmed the anti-inflammatory activities of total triterpenoids in *A. arguta* branches, which can effectively reduce the production and development of inflammation and maintain normal physiological functions [79]. However, the mechanism of its anti-inflammatory effect still needs to be studied. The anti-inflammatory effects of the fruit of *A. arguta* were investigated using an LPS-stimulated RAW 264.7 murine macrophage cell line. The polyphenols and flavonoids in the fruit of *A. arguta* can effectively inhibit the release of interleukin-6 (IL-6) and tumor necrosis factor-*α* (TNF-*α)*, and their effects on the release of NO are dose-dependent. Therefore, inhibition of this pathway could be a possible mechanism of the anti-inflammatory effects of the fruit of *A. arguta* [63].

### 4.3. Anti-Tumor Activity

The anti-tumor effect is mainly reflected in inhibiting the proliferation and growth of tumor cells, promoting tumor cell apoptosis, enhancing the body’s immunity, and alleviating symptoms. *A. arguta* exhibits a significant anti-tumor effect, with its roots, stems, leaves, and fruit containing various anti-tumor active ingredients, such as flavonoids, anthraquinones, and polysaccharides. A mouse model of reduced bone marrow function was created using a single intravenous injection of 5-fluorouracil at a dose of 150 mg/kg. Methanol extract from *A. arguta* stems (100 mg/kg/d) promotes the proliferation of mouse bone marrow cells, in which (+)-catechin and (−)-epi-catechin play a role [80]. (+)-catechin (1 and 10 mg/kg/d) was found to be effective in promoting bone marrow cell proliferation and combating the hematotoxicity of 5-fluorouracil in mice [81]. Five types of human cancer cells were cultured in Dulbecco’s Modified Eagle Medium (DMEM) supplemented with 10% fetal bovine serum in a 5% CO_2_-humified incubator at 37 °C. It was observed that *A. arguta* fruit exhibits anti-proliferative activities against Hep3B and HeLa cell lines but has no effect on HT-29, HepG2, and LoVo cells [82]. The inhibitory effect of extracts on leukemia cells was detected using the 3-(4,5-dimethylthiazol-2-yl)-2,5-diphenyl-2H-tetrazolium bromide (MTT) assay.

Anthraquinone compounds extracted from *A. arguta* roots exhibited significant inhibitory effects on four leukemia cell lines, namely, JARKET, RAJI, L1210, and K562. The inhibitory rate shows a positive correlation with the concentration of the extract [83]. Furthermore, the polysaccharide derived from *A. arguta* stems (20 mg/mL) inhibited the proliferation of transplanted S180 tumor cells in Swiss mice, and this anti-tumor effect might be attributed to the enhanced immune function of the body [84]. *A. arguta* juice has the ability to block the formation of *N*-nitroso morpholine (a known carcinogen) under simulated gastric juice conditions, with a blocking rate of 79.52%. This rate is higher than that of an equivalent amount of vitamin C solution, indicating the strong anti-tumor effects of *A. arguta* juice [85]. The volatile components of *A. arguta* also exhibit cytotoxic activities. The cytotoxicity of *A. arguta* essential oil was studied using the MTT assay, yielding IC_50_ values of 6.067 mg/mL, 11.905 mg/mL, and 13.646 mg/mL for the A549, HT-29, and PC-3 cell lines, respectively [46]. The antiproliferative activities of *A. arguta* extract against HepG2 and HT-29 cells (0–100 mg/mL and 0–200 mg/mL, respectively) were found to be 1.44–4.25 times higher than that of *Actinidia kolomikta* or *Actinidia chinensis* extracts, likely due to the higher flavonoid content in *A. arguta* extract. Therefore, *A. arguta* extract shows potential as a chemotherapeutic agent against HepG2 and HT-29 cells [86].

### 4.4. Anti-Aging Activity

Modern medicine recognizes skin aging as a physiological or pathological change influenced by various factors and is categorized as either endogenous aging or exogenous aging [87]. Endogenous aging is influenced by genetic or endocrine factors, while exogenous aging is influenced by external environmental factors that can lead to skin laxity, roughness, and deepening wrinkles [88]. Traditional Chinese medicine contains active ingredients, such as saponins, polysaccharides, and flavonoids, that possess anti-aging effects by scavenging free radicals, regulating immunity, reducing mitochondrial DNA damage, improving substance metabolism, and enhancing microcirculation [89].

In one study, 14-month-old TA_1_ pure-strain mice were fed *A. arguta* juice for 50 d [90]. The results showed that male and female mice had 75.17% and 76.25% inhibition rates of MAO-B activity in the brain, indicating that *A. arguta* juice can modulate the central aging clock by regulating brain monoamine levels and exerting anti-aging effects. *A. arguta* juice also significantly reduced lipofuscin content in mouse myocardial cells, demonstrating its antioxidant effect and ability to inhibit free radical production. Moreover, it reduced the hydroxyproline content in the tail tendons of 14-month-old TA_1_ pure-strain mice, suggesting its potential to delay aging. Additionally, *A. arguta* juice decreased the levels of total bile acid (TBA) and increased SOD activity. However, further research is needed to determine the specific mechanisms behind its anti-aging effects [91]. In another study, Gan et al. (2004) administered *A. arguta* juice to elderly Wistar rats aged 20–22 months with a weight of 400 ± 30 g at doses of 3 g/kg and 6 g/kg for 30 d. The measurements of red blood cell and liver SOD, whole blood glutathione peroxidase (GSH-Px) activity, serum MDA, and lipofuscin content in brain and heart tissues revealed a significant increase in red blood cell SOD, liver SOD, and whole blood GSH-Px activity. Additionally, the levels of serum MDA and lipofuscin in brain and heart tissues were significantly reduced. These findings indicate that *A. arguta* juice could enhance the activities of antioxidant enzymes, reduce lipid peroxidation, and delay the aging process in the bodies of elderly rats [92].

### 4.5. Anti-Fatigue Effect

Effervescent tablets were produced using *A. arguta* fruit through spray drying and tablet pressing technologies in certain studies. The dose was divided into low-, medium-, and high-dose groups, with an administration of 0.2 mL per 20 g weight of medication for 20–22 g Kunming male mice. Compared with the control group, the effervescent tablet low-, medium-, and high-dose groups exhibited significant differences in terms of prolonged exhaustive swimming time in mice, with increases of 35.03%, 61.15%, and 89.81%, respectively. Similarly, the fatigue rotation time of mice increased by 58.10%, 122.86%, and 157.14% in the low-, medium-, and high-dose groups, respectively. While the low-dose group displayed a significant difference, the medium- and high-dose groups showed extremely significant differences. Furthermore, effervescent tablets were found to enhance exercise endurance and improve parameters such as lactate dehydrogenase activity, liver glycogen, and muscle glycogen content in mice. Additionally, they reduced serum urea nitrogen and blood lactate content, demonstrating significant anti-fatigue effects [93]. In another experiment, 28 Kunming mice were randomly allocated into four groups and administered doses of 0, 50, 100, and 200 mg/kg/d of crude alkaloid extract from *A. arguta* for 28 d raised in an SPF barrier system. The evaluation of exercise abilities included forelimb strength training and weight-bearing swimming time, while anti-fatigue abilities were assessed by measuring the glycogen content in the liver and muscles, as well as observing morphological changes in the longitudinal profiles of striated and skeletal muscles. The results indicated that the crude alkaloid extract improved the endurance and grip strength of mice and prolonged their swimming time under load, with the 100 mg/kg/d group displaying the most significant prolongation. Compared with the control group, the experimental group showed significant decreases in the levels of lactate, ammonia, and creatine kinase, accompanied by an increase in tissue glycogen content. Moreover, no changes were observed in the morphology of striated and skeletal muscles [94].

### 4.6. Hypoglycemic Activity

Plant polysaccharides have the beneficial effect of reducing blood sugar levels. They are safe, with minimal toxic and side effects, and show positive effects in individuals with hyperglycemia. As a result, an increasing number of researchers are dedicated to utilizing plant polysaccharides in the development of safe, affordable, and effective natural medications for blood sugar reduction [95,96]. *A. arguta* polysaccharides exhibit hypoglycemic effects, and studies indicate that the main active compounds responsible for this effect in *A. arguta* are flavonoids and polysaccharides [97,98]. In one study, mice induced with type II diabetes through a high-fat and high-sugar diet, combined with streptozocin (STZ), were administered low, medium, and high doses of *A. arguta* flavonoids at 90, 180, and 270 mg/kg/d, respectively. The results show a significant improvement in the symptoms of the diabetic mice, specifically in reduced fasting blood glucose levels and serum insulin levels. The fasting blood glucose level decreased correspondingly as the dose increased. Additionally, it was observed that the expression level of the glucokinase gene in the liver of the diabetic mice increased, thereby enhancing the glucokinase activity. The hypoglycemic effect of flavonoids on type II diabetic mice is thought to be mediated by upregulating the expression of the glucokinase gene, repairing damaged pancreatic islet *β* cells, improving serum insulin levels, inhibiting *α*-glucosidase activity, and enhancing glucokinase activity, thus maintaining glucose homeostasis in the liver [98].

The polyphenol fraction of *A. arguta*, which contains quercetin-3-O-glucoside and quercetin-3-O-galactoside, exhibits inhibitory activity against *α*-glucosidase and maltase. Male KK-A^y^ mice, which is a type II diabetic model, and C57BL/6J mice, which is a non-diabetic control model of KK-A^y^ mice, in addition to male Sprague Dawley (SD) rats, were used for a single-dose test. In an oral glucose tolerance test conducted on KK-A^y^ mice fed with quercetin-3-O-glucoside for 4 weeks, blood glucose levels tended to be lower 60 min after glucose administration. These findings suggest that *A. arguta* possesses antidiabetic effects, and quercetin-3-O-glucoside, which is a component of *A. arguta*, may be useful in the prevention of type II diabetes by suppressing gluconeogenesis and enhancing lipid *β*-oxidation [99].

To explore the hypoglycemic effect of polysaccharides, a model of type II diabetic mice was established by feeding them with a high-fat and high-sugar diet and intraperitoneal injection of a low-dose STZ. Polysaccharides derived from *A. arguta* branches were orally administered to mice in low-, medium-, and high-dose groups at 10, 20, and 40 mg/kg, respectively. Another group of mice was administered 40 mg/kg of metformin as a positive control. After 28 d of continuous administration, there was a significant decrease in total cholesterol, total triglycerides, and low-density lipoprotein cholesterol, while high-density lipoprotein cholesterol levels significantly increased. This indicates an improvement in the abnormal blood lipid profile found in diabetic mice. Moreover, the content of MDA in serum significantly decreased, while the content of SOD increased, suggesting that polysaccharides may have a hypoglycemic effect by inhibiting peroxidation reactions in the body. An examination of organ indices and histomorphology revealed that the high-dose polysaccharide group exhibited a protective effect on the pancreas and liver of STZ-induced diabetic mice. Therefore, it can be inferred that polysaccharides regulate blood sugar levels and lipid metabolism in diabetic mice while also reducing insulin resistance [100].

*A. arguta* fruit polysaccharide has a reparative effect on the islets of the pancreas in diabetic mice induced by intraperitoneal injection of alloxan. It increases insulin secretion, improves glucose and lipid metabolism, significantly reduces fasting blood sugar, increases glucose tolerance, boosts liver glycogen, and lowers blood lipid levels [101]. However, the exact mechanism still requires further exploration. Compared with the control group, rats with STZ-induced diabetes that were fed with the 70% ethanol extract (400 mg/kg) of *A. arguta* experienced a significant reduction in postprandial blood glucose by inhibiting *α*-glucosidase activity [102]. Additionally, in a study using mice fed with a high-fat and high-sugar diet, an extract of *A. arguta* not only reduced barbituric acid reactive substances but also increased GSH levels [103]. Another study showed that a polyphenol extract in the fruit of *A. arguta* also inhibited *α*-glucosidase activity [103]. Furthermore, an aqueous extract of *A. arguta* stem exhibited appreciable inhibitory activity against the *α*-glucosidase enzyme, with an IC_50_ of 1.71 mg/mL [68].

### 4.7. Hypolipidemic Activity

In recent years, the incidence rate of hyperlipidemia has been increasing. Hyperlipidemia is a sign of a lipid metabolism disorder and one of the main triggers for atherosclerosis, heart disease, and fatty liver [104]. Preventing the occurrence and development of hyperlipidemia is of great significance for reducing the incidence rate of cardiovascular and cerebrovascular diseases [104]. The search for lipid-lowering drugs from natural plants is currently a prominent research topic [105,106]. Leontowicz et al. (2016) investigated the protective effects of *A. arguta* fruit on the aorta and liver of hypercholesterolemic rats. They fed 71 male Wistar rats with 1% cholesterol to induce a model and then randomly divided them into groups. In the *A. arguta* group, the levels of total cholesterol, low-density lipoprotein cholesterol, the total cholesterol/high-density lipoprotein cholesterol atherosclerosis index, and triglyceride in the liver serum were reduced, accompanied by an increase in high-density lipoprotein cholesterol. Additionally, the serum’s antioxidant capacity became stronger. Rat liver fibrosis decreased, the prothrombin time was prolonged, and serum peroxidase decreased. This suggests that *A. arguta* can effectively reduce the probability of hyperlipidemia [107].

There are also reports of a study that selected 36 male mice and randomly divided them into three groups: blank control, high-fat model, and high-fat+polysaccharide groups, each containing 12 mice. The high-fat+polysaccharide group was fed a high-fat diet daily, while *A. arguta* fruit polysaccharide (300 mg/kg) was administered via gavage. The results demonstrated that polysaccharides significantly reduced the content of total cholesterol and triglycerides in both the serum and liver of mice while increasing the level of high-density lipoprotein cholesterol in the liver. Pathological observations of liver tissue indicated that polysaccharides significantly improved and alleviated the symptoms of liver fat and hyperlipidemia. Determining the lipid levels in mouse feces revealed that polysaccharides significantly increased the total fat, cholesterol, and triglyceride contents in the feces of high-fat mice, suggesting that the polysaccharide’s effect on reducing cholesterol levels is achieved by promoting the cholesterol excretion pathway [108].

### 4.8. Other Pharmacological Effects

*A. arguta* also exhibits other pharmacological effects, such as antibacterial, antiglycated, anti-radiation, and immune-regulation properties. Studies have reported the in vitro antibacterial activity of *A. arguta*. The results indicate that *A. arguta* volatile oil has significant antibacterial activity against *Staphylococcus aureus* and *Saccharomyces cerevisiae*, with inhibition zones of 19.5 mm and 20.5 mm, respectively. The antibacterial activities against *Bacillus subtilis* and *Microsporum canis* are moderate, with inhibition zones of 17.2 mm and 16.8 mm, respectively. However, *A. arguta* shows weak antibacterial activities against *Escherichia coli* and *Pseudomonas aeruginosa*, with inhibition zones of 8.5 mm and 10 mm, respectively [46]. Additionally, studies indicate that *A. arguta* fruit polysaccharides have inhibitory effects on *Bacillus subtilis*, *Escherichia coli*, *Staphylococcus aureus*, and other bacteria. The antibacterial effect increases as the concentration of polysaccharides increases, with a minimum inhibitory concentration (MIC) ranging from 10 to 25 mg/mL. However, no antibacterial effect was observed against *Candida tropicalis*. Temperature and pH values also impact the antibacterial effect of polysaccharides, with a higher temperature resulting in a more pronounced effect. Polysaccharides exhibit a better antibacterial effect within a pH range of 4–5 [109]. The total flavonoids of *A. arguta* have inhibitory effects on *Escherichia coli*, *Staphylococcus aureus*, *Rhizopus*, *Aspergillus oryzae*, *Candida tropicalis*, and *Saccharomyces cerevisiae*. The inhibitory effect increases as the concentration of total flavonoids increases, with an MIC ranging from 25 to 50 mg/mL. No inhibitory effect was observed against *Aspergillus niger*. Similar to polysaccharides, temperature and pH values also affect the antibacterial effect of total flavonoids, with a higher temperature having a more pronounced effect. Total flavonoids exhibit a better antibacterial effect within a pH range of 5–6 [110]. Another study investigated the antibacterial activity of *A. arguta* fruit and leaves. The crude extracts of *A. arguta* fruit and leaves notably inhibited clinical strains of *Pseudomonas aeruginosa* and *Escherichia coli*, which were resistant to the action of ofloxacin. The inhibitory effects of the plant extracts on clinical strains of *Klebsiella pneumoniae* and *Acinetobacter baumannii* were comparable with the effect of ofloxacin [70]. In addition, Macedo et al. (2023) studied an extract of *A. arguta*, which displayed antimicrobial activities against *Staphylococcus aureus* (MIC = 32 mg/mL) and *Pseudomonas gingivalis* (MIC = 64 mg/mL) and reduced the growth rate of *Escherichia coli* [71].

A novel cell wall polysaccharide (AAPs) was extracted from *A. arguta* fruit and separated into four parts, namely, water-eluted polysaccharide, salt-eluted polysaccharide (SPS)-1, SPS-2, and SPS-3. All four types of polysaccharides exhibited the ability to scavenge free radicals, chelate iron ions, inhibit lipid peroxidation, and inhibit protein glycation. However, SPS showed significantly stronger effects compared with the water-eluted polysaccharide. Particularly, SPS-3 demonstrated the highest antioxidant and anti-glycated activities. Furthermore, the inhibitory effect of AAPs on advanced glycation end product formation can be attributed to their ability to inhibit the production of protein carbonyl groups and protect protein thiol groups. This effect is not related to the scavenging capacity of dicarbonyl compounds. These findings suggest that the mechanisms underlying the antiglycated effects of AAPs may be linked to their antioxidant activities [111]. Moreover, more than 20% of *A. arguta* fruit polysaccharides significantly enhanced the survival rate of yeast cells after ultraviolet radiation, indicating the radiation-protective effects of these polysaccharides on cells [112].

Studies also demonstrated that *A. arguta* stem polysaccharides have a notable immune-promoting effect and act as effective immune regulators in mice (20 ± 2 g). These polysaccharides enhance the proliferation of T and B lymphocytes both in vivo and in vitro, with the most significant effect observed at a dose of 100 mg/kg. Additionally, they promote mitosis and stimulate the production of lymphokines in mice. Furthermore, *A. arguta* stem polysaccharides enhance the primary response of B cells to SRBC antibodies and improve the phagocytic ability of macrophages [113]. Other research indicates that *A. arguta* fruit polysaccharides briefly increase the proportion of total T cells and helper T cells in 6-week-old female mouse peripheral blood, have a long-term inhibitory effect on the proportion of B cells and toxic T cells in mouse peripheral blood, and exhibit delayed and instantaneous promotion of the proportion of NK cells in mouse peripheral blood [114]. Moreover, high doses of *A. arguta* fruit polysaccharides promote the growth of SD rats and significantly increase their spleen index. The medium- and high-dose groups also exhibit significant increases in the thymic index, phagocytic index, and concanavalin A (Con A)-induced splenic lymphocyte transformation index in rats. These findings confirm that polysaccharides enhance the immune system by promoting the growth of immune organs, enhancing cellular immune function, and improving the phagocytic ability of monocytes and macrophages [115]. In terms of safety, three solvent extracts (water, water:ethanol (50:50), and ethanol) of *A. arguta* leaves showed no adverse effects on caco-2 and HT29-MTX cells at concentrations below 100 μg/mL and 1000 μg/mL, respectively. This suggests that *A. arguta* leaves are relatively safe for consumption [116].

## 5. Discussion

We conducted a comprehensive review of the phytochemistry and pharmacological research of *A. arguta*, which is a traditional medicinal plant. A total of 539 compounds were reported, showing that *A. arguta* contains a variety of phytochemicals, including terpenoids, flavonoids, phenolics, phenylpropanoids, lignin, organic acids, volatile components, alkanes, coumarins, anthraquinones, alkaloids, polysaccharides, and inorganic elements. We also elucidated the various pharmacological studies on these compounds and various extracts of *A. arguta*. This thorough literature review indicates that *A. arguta* has excellent antioxidant, anti-inflammatory, and anti-tumor properties. Furthermore, it has broad application prospects for improving glucose metabolism, anti-aging, anti-fatigue, and immune regulation. In particular, flavonoids, phenolics, and polysaccharides, which were identified as the main components responsible for mediating these pharmacological effects, were extensively studied and reported multiple times.

Additionally, the roots of *A. arguta* have long been known for their unique anticancer effects and are highlighted in the “Dietary Materia Medica”, along with the “Compendium of Materia Medica”. Therefore, a significant amount of academic research is dedicated to revealing the active ingredient of its anticancer efficacy. In recent years, *A. arguta*, as a new fruit, has gradually been accepted by more and more people due to its excellent taste and rich nutritional value. It is used to prepare jam, canned food, and wine, making it an emerging resource for research and processing in the food industry. This has driven a surge in demand in both domestic and international markets, leading to the large-scale planting of *A. arguta*. Consequently, researchers have been inspired to explore the different medicinal parts of the plant and strive to broaden their research scope. Amongst these sections, the leaves of *A. arguta* have received attention due to their unique characteristics similar to tea. However, current research on the chemical composition of *A. arguta* mainly focuses on the isolation and identification of individual compounds, with limited research on the changes in component content in different regions and plant parts. Additionally, further exploration is needed to establish quality standards for *A. arguta*. It is important to highlight future research in these areas, as it is crucial for enhancing the standardized application and quality control of *A. arguta*.

Of particular interest in *A. arguta* are flavonoids and phenolics. Researchers discovered a total of 52 compounds from these categories in *A. arguta*, which showed extensive clinical efficacy in clinical studies regarding anti-aging and hypoglycemic effects. It is noteworthy that *A. arguta* contains compounds with specific structures, such as (2*R*,6*R*,9*R*)-trihydroxy-megastigmane-4,7*E*-dien-3-one-9-O-*β*-D-glucopyranoside, which is a methylcyclohexene-type sesquiterpenoid glycoside compound that has not been reported in the literature. These unique chemical characteristics make *A. arguta* a promising subject for further exploration of the biological activity of these novel terpenoids and the potential discovery of safe and effective compounds with therapeutic applications.

Moreover, numerous studies revealed the various pharmacological activities of *A. arguta*, mainly focusing on its antioxidant, anti-inflammatory, and anti-tumor effects. However, it is important to note that some studies only utilize extracts from different parts or solvents of *A. arguta*, rather than pure compounds. Additionally, the research on other pharmacological activities of *A. arguta* is not comprehensive enough, with many mechanisms of action remaining unidentified. Currently, research on the pharmacokinetics of *A. arguta* is limited, despite its significant role in elucidating metabolic pathways. Therefore, further exploration of the pharmacological activity and pharmacokinetics of *A. arguta* is necessary.

This review, however, has certain limitations. First, the methods used for collecting literature and data were limited, which may lead to the omission of relevant studies. Second, the quality of some collected research literature may have flaws, potentially affecting the reliability of the review’s results. Another significant limitation was the lack of research on the toxicity and clinical reports of *A. arguta*, especially regarding its significant therapeutic effect on gastrointestinal tumors. Further studies should address these limitations and delve into unexplored areas, such as toxicity, pharmacokinetics, and clinical research.

## 6. Conclusions

However, there is currently a dearth of comprehensive and detailed documentation on the phytochemistry and pharmacology of *A. arguta*. As a result, the main objective of this review was to thoroughly explore the existing research on *A. arguta* by examining multiple databases and addressing these aforementioned aspects. Additionally, this review aimed to establish a strong foundation for further exploration of the potential uses of *A. arguta*, as well as providing guidance for future research. First, it was demonstrated that the roots of *A. arguta* exhibit remarkable efficacy as an anti-tumor agent, particularly in the treatment of gastrointestinal tumors. This finding suggests its potential as a valuable addition to the arsenal of anti-cancer drugs. Second, given the deep-rooted love for tea culture in East Asian countries, products such as health teas derived from the leaves of *A. arguta* hold tremendous potential for development. These teas could cater to the growing demand for natural and beneficial beverages, offering unique flavors and potential health benefits. Furthermore, the fruit of *A. arguta* is not only nutritionally rich but also boasts an appealing taste, making it increasingly popular among consumers. As awareness of the fruit’s health benefits spreads, it is expected that more and more individuals will embrace and enjoy the fruit of *A. arguta*. Building upon this extensive review, further investigations into *A. arguta* are likely to lead to the isolation and identification of additional chemical components. Additionally, ongoing research will undoubtedly uncover more effective and practical pharmacological effects, ultimately benefiting humanity and contributing to the advancement of medical science.

## Figures and Tables

**Table 1:** Terpenoids isolated from *Actinidia arguta*.

No.	Name	Formula	Exact Theoretical Molecular Weight	Source	Characterization Method	Refs.
1	3*β*-Hydroxyurs-12-en-28-oic acid	C_30_H_48_O_3_	456.3603	Leaves	EI-MS, ^1^H-NMR, ^13^C-NMR	[16]
2	3*β*,24-Dihydroxyurs-12-en-28-oic acid	C_30_H_48_O_4_	472.3553	Leaves	IR, EI-MS, ^1^H-NMR, ^13^C-NMR	[16]
3	2*α*,3*α*,24-Trihydroxyurs-12-en-28-oic acid	C_30_H_48_O_5_	488.3502	Leaves	IR, EI-MS, ^1^H-NMR	[17]
4	2*α*,3*β*-Dihydroxyurs-12-en-28,30-olide	C_30_H_46_O_4_	470.3396	Roots	HPLC-DAD-ESI-MS	[18]
5	12*α*-Chloro-2*α*,3*β*,23-tetrahydroxyolean-28-oic acid-13-lactone	C_30_H_47_O_5_Cl	522.3112	Roots	HPLC-DAD-ESI-MS	[18]
6	Oleanolic acid	C_30_H_48_O_3_	456.3603	Roots	HPLC	[14,15]
7	Ursolic acid	C_30_H_48_O_3_	456.3603	Roots, stems	IR, MS, ^13^C-NMR	[14,15,19]
8	Acetyl oleanolic acid	C_32_H_50_O_4_	4983709	Stems	IR, MS, ^1^H-NMR, ^13^C-NMR	[19]
9	Actinidic acid	C_30_H_46_O_5_	486.3345	Leaves	UV, IR, HR-ESI-TOF-MS, ^1^H-NMR, ^13^C-NMR, HSQC, HMBC, NOESY	[20]
10	Actiniargupene A	C_30_H_46_O_4_	470.3396	Leaves	UV, IR, HR-ESI-TOF-MS, ^1^H-NMR, ^13^C-NMR, HSQC, HMBC, NOESY	[20]
11	Actiniargupene B	C_39_H_52_O_7_	632.3713	Leaves	UV, IR, HR-ESI-TOF-MS, ^1^H-NMR, ^13^C-NMR, HSQC, HMBC, NOESY	[20]
12	Actiniargupene C	C_39_H_52_O_7_	632.3713	Leaves	UV, IR, HR-ESI-TOF-MS, ^1^H-NMR, ^13^C-NMR, HSQC, HMBC, NOESY	[20]
13	3-O-*trans*-*p*-Coumaroyl actinidic acid	C_39_H_52_O_7_	632.3713	Leaves	IR, HR-ESI-TOF-MS, ^1^H-NMR, ^13^C-NMR, HSQC, HMBC, NOESY	[20]
14	3-O-*cis*-*p*-Coumaroyl actinidic acid	C_39_H_52_O_7_	632.3713	Leaves	IR, HR-ESI-TOF-MS, ^1^H-NMR, ^13^C-NMR, HSQC, HMBC, NOESY	[20]
15	2*α*,3*α*,23-Trihydroxyursa-12,20(30)-dien-28-oic acid	C_39_H_52_O_7_	632.3713	Leaves	IR, HR-ESI-TOF-MS, ^1^H-NMR, ^13^C-NMR, HSQC, HMBC, NOESY	[20]
16	Dehydroisoactinidic acid	C_30_H_46_O_5_	486.3345	Leaves	UV, IR, HR-ESI-TOF-MS, ^1^H-NMR, ^13^C-NMR, HSQC, HMBC, NOESY	[20]
17	Actiniargupene D	C_39_H_54_O_7_	634.3870	Leaves	UV, IR, HR-ESI-TOF-MS, ^1^H-NMR, ^13^C-NMR, HSQC, HMBC, NOESY	[20]
18	Actiniargupene E	C_39_H_54_O_8_	650.3819	Leaves	UV, IR, HR-ESI-TOF-MS, ^1^H-NMR, ^13^C-NMR, HSQC, HMBC, NOESY	[20]
19	Actiniargupene F	C_39_H_54_O_7_	634.3870	Leaves	UV, IR, HR-ESI-TOF-MS, ^1^H-NMR, ^13^C-NMR, HSQC, HMBC, NOESY	[20]
20	3-O-*trans*-*p*-Coumaroylasiatic acid	C_39_H_54_O_7_	634.3870	Leaves	UV, IR, HR-ESI-TOF-MS, ^1^H-NMR, ^13^C-NMR, HSQC, HMBC, NOESY	[20]
21	23-O-*trans*-*p*-Coumaroylasiatic acid	C_39_H_54_O_7_	634.3870	Leaves	UV, IR, HR-ESI-TOF-MS, ^1^H-NMR, ^13^C-NMR, HSQC, HMBC, NOESY	[20]
22	11*α*-Methoxyurs-12-ene-3*β*,12-diol	C_31_H_52_O_3_	472.3916	Leaves	UV, IR, HR-ESI-TOF-MS, ^1^H-NMR, ^13^C-NMR, HSQC, HMBC, NOESY	[20]
23	Ilelatifol A	C_30_H_48_O_3_	456.3603	Leaves	UV, IR, HR-ESI-TOF-MS, ^1^H-NMR, ^13^C-NMR, HSQC, HMBC, NOESY	[20]
24	(2*R*,6*R*,9*R*)-Trihydroxy-megastigmane-4,7*E*-dien-3-one-9-O-*β*-D-glucopyranoside	C_19_H_30_O_9_	402.1890	Fruit	UV, ^1^H-NMR, ^13^C-NMR, HRESI-TOF-MS, HMBC, NOESY, HPLC, ECD	[21]
25	(6*S*,9*R*)-Roseoside	C_19_H_30_O_8_	386.0941	Fruit	ESI-MS, ^1^H-NMR, ^13^C-NMR, ECD	[21]

UV: ultraviolet spectrophotometry; IR: infrared spectroscopy; ^13^C-NMR: carbon-13 nuclear magnetic resonance spectrometry; ^1^H-NMR: hydrogen-1 nuclear magnetic resonance spectrometry; ESI-MS: electrospray ionization-mass spectrometry; MS: mass spectrometry; HMBC: ^1^H-detected heteronuclear multiple bond correlation; NOESY: nuclear Overhauser effect spectroscopy; HPLC: high-performance liquid chromatography; ECD: electrical conductivity detector; HRESI-TOF-MS: high-resolution electrospray ionization-time of flight-mass spectrometry; HPLC-DAD-ESI-MS: high-performance liquid chromatography-diode array detection-electrospray ionization-mass spectrometry.

**Table 2:** Flavonoids isolated from *Actinidia arguta*.

No.	Name	Formula	Exact Theoretical Molecular Weight	Source	Characterization Method	Refs.
26	Rutin	C_27_H_30_O_16_	610.1534	Skin and flesh of the ripe fruit	HPLC	[22]
27	Quercetin	C_15_H_10_O_7_	302.0427	Skin and flesh of the ripe fruit	HPLC	[22]
28	(−)-*epi*-Catechin	C_15_H_14_O_6_	290.0790	Roots	ESI-MS, ^1^H-NMR, ^13^C-NMR	[25]
29	(+)-Catechin	C_15_H_14_O_6_	290.0790	Roots	ESI-MS, ^1^H-NMR, ^13^C-NMR	[25]
30	Proanthocyanidin B2	C_30_H_26_O_12_	578.1424	Fruit	UV, HPLC-MS	[24]
31	Proanthocyanidin C1	C_45_H_38_O_18_	866.2058	Fruit	UV, HPLC-MS	[24]
32	(+)-Gallocatechin	C_15_H_14_O_7_	306.0740	Fruit	UV, HPLC-MS	[24]
33	Quercetin-3-O-galactoside	C_21_H_20_O_12_	464.0955	Fruit	UV, HPLC-MS	[24]
34	Quercetin-3-O-rutinoside	C_27_H_30_O_16_	610.1534	Fruit	UV, HPLC-MS	[24]
35	Quercetin-3-O-glucoside	C_21_H_20_O_12_	464.0955	Fruit	UV, HPLC-MS	[24]
36	Astragalin	C_21_H_20_O_11_	448.1006	Fruit	ESI-MS, ^1^H-NMR, ^13^C-NMR	[21]
37	Procyanidin B4	C_30_H_26_O_12_	578.1424	Roots	ESI-MS, ^1^H-NMR, ^13^C-NMR	[25]
38	6-(2-Pyrrolidinone-5-yl)-(−)-epicatechin	C_19_H_19_NO_7_	373.1162	Roots	IR, ESI-MS, HR-ESI-MS, ^1^H-NMR, ^13^C-NMR, HMBC	[25]
39	8-(2-Pyrrolidinone-5-yl)-(−)-epicatechin	C_19_H_19_NO_7_	373.1162	Roots	IR, FAB-MS, HR-FAB-MS, ^1^H-NMR, ^13^C-NMR, HMBC	[25]
40	Kaempferol-3-O-rutinoside (+)	C_27_H_30_O_15_	594.1585	Fruit	LC-MS/MS	[23]
41	Kaempferol-3-O-rutinoside (−)	C_27_H_30_O_15_	594.1585	Fruit	LC-MS/MS	[23]
42	Kaempferol-3-O-neohesperidoside	C_27_H_30_O_15_	594.1585	Fruit	LC-MS/MS	[23]
43	Isorhamnetin-3-O-neohesperidoside (+)	C_28_H_32_O_16_	624.1690	Fruit	LC-MS/MS	[23]
44	Isorhamnetin-3-O-neohesperidoside (−)	C_28_H_32_O_16_	624.1690	Fruit	LC-MS/MS	[23]
45	Isorhamnetin-3-O-rutinoside	C_28_H_32_O_16_	624.1690	Fruit	LC-MS/MS	[23]
46	Isorhamnetin-3-O-neohespeidoside	C_28_H_32_O_16_	624.1690	Fruit	LC-MS/MS	[23]
47	Quercetin-3-O-rhamnoglucoside	C_25_H_28_O_15_	568.1428	Fruit	LC-MS/MS	[23]
48	Quercetin-3-O-*β*-D-galactopyranoside	C_21_H_20_O_12_	464.0955	Fruit	ESI-MS, ^1^H-NMR, ^13^C-NMR	[21]
49	Quercetin-3-O-[*α*-rhamnopyranosyl-(1-4)-rhamnopyranosyl-(1-6)-*β*-galactopyranoside	C_37_H_48_O_20_	812.2739	Plant material	UV, MS, ^1^H-NMR, ^13^C-NMR	[26]
50	Kaempferol-3-O-[*α*-rhamnopyranosyl-(1-4)-rhamnopyranosyl-(1-6)-*β*-galactopyranoside	C_37_H_48_O_19_	796.2790	Plant material	^1^H-NMR, ^13^C-NMR	[26]
51	Quercetin 3-sambubioside	C_26_H_28_O_16_	596.1377	Leaves	PC, GC, UV, ^1^H-NMR, ^13^C-NMR	[27]
52	Quercetin 3-O-*β*-D-[2-O-*β*-D-xylopyranosy-6-O-*α*-L-rhamnopyranosyl] glucopyranoside	C_32_H_38_O_20_	742.1956	Leaves	PC, GC, UV, ^1^H-NMR, ^13^C-NMR	[27]
53	Isorhamnetin-3-O-*α*-L-rhamnopyranosyl-(1-3)-*α*-L-rhamnopyranosyl-(1-6)-*β*-D-galactopyranoside	C_34_H_42_O_20_	770.2269	Fruit	LC-MS/MS	[23]

UV: ultraviolet spectrophotometry; IR: infrared spectroscopy; PC: paper chromatography; GC: gas chromatography; ^13^C-NMR: carbon-13 nuclear magnetic resonance spectrometry; ^1^H-NMR: hydrogen-1 nuclear magnetic resonance spectrometry; ESI-MS: electrospray ionization-mass spectrometry; MS: mass spectrometry; HMBC: ^1^H-detected heteronuclear multiple bond correlation; HPLC: high-performance liquid chromatography; LC-MS/MS: liquid chromatography-mass spectrometry/mass spectrometry.

**Table 3:** Phenolic compounds isolated from *Actinidia arguta*.

No.	Name	Formula	Exact Theoretical Molecular Weight	Source	Characterization Method	Refs.
54	Planchol A	C_14_H_14_O_6_	278.0790	Roots	HPLC-DAD-ESI-MS	[18]
55	Planchol B	C_15_H_16_O_6_	292.0947	Roots	HPLC-DAD-ESI-MS	[18]
56	Isotachioside	C_13_H_18_O_8_	302.1002	Roots	HPLC-DAD-ESI-MS	[18]
57	*p*-Hydroxybenzoic acid	C_7_H_6_O_3_	138.0317	Roots, leaves	ESI-MS, ^1^H-NMR, ^13^C-NMR	[25,29]
58	Vanillic acid	C_8_H_8_O_4_	168.0423	Roots, leaves	EI-MS, ^1^H-NMR, ^13^C-NMR	[29]
59	Protocatechuic acid	C_7_H_6_O_4_	154.0266	Leaves, fruit	IR, HRESI-TOF-MS, ^1^H-NMR, ^13^C-NMR, HMBC	[21,29]
60	Isovanillic acid	C_8_H_8_O_4_	168.0423	Leaves	IR, HRESI-TOF-MS, ^1^H-NMR, ^13^C-NMR, HMBC	[29]
61	Hydroxytyrosol	C_12_H_16_O_7_	154.0630	Leaves	IR, HRESI-TOF-MS, ^1^H-NMR, ^13^C-NMR, HMBC	[29]
62	Caffeoylthreonic acid	C_12_H_16_O_7_	298.0689	Leaves	HPLC-MS/MS	[30]
63	salvianic acid A	C_10_H_12_O_5_	212.0685	Leaves	HPLC-MS/MS	[30]
64	Maysedilactone A	C_15_H_16_O_8_	324.0845	Leaves	IR, ESI-MS, HR-ESI-MS, ^1^H-NMR, ^13^C-NMR	[31]
65	Maysedilactone D	C_15_H_16_O_9_	340.0794	Leaves	IR, ESI-MS, HR-ESI-MS ^1^H-NMR, ^13^C-NMR, HMBC, NOESY	[31]
66	Maysedilactone B	C_16_H_18_O_9_	354.0951	Leaves	IR, ESI-MS, HR-ESI-MS, ^1^H-NMR, ^13^C-NMR	[31]
67	Argutinoside J	C_18_H_22_O_11_	414.1162	Fruit	HRESI-TOF-MS, IR, UV, ^1^H-NMR, ^13^C-NMR, HMBC	[32]
68	Argutinoside K	C_19_H_24_O_12_	444.1268	Fruit	HRESI-TOF-MS, IR, UV, ^1^H-NMR, ^13^C-NMR, HMBC	[32]
69	Argutinoside L	C_20_H_28_O_10_	428.1682	Fruit	HRESI-TOF-MS, IR, UV, ^1^H-NMR, ^13^C-NMR, HMBC	[32]
70	Vanillic acid-4-O-*β*-D-glucopyranoside	C_14_H_18_O_9_	330.0951	Fruit	ESI-MS, ^1^H-NMR, ^13^C-NMR	[21]
71	1-O-Feruloyl-*β*-D-glucopyranoside	C_16_H_20_O_9_	356.1107	Fruit	ESI-MS, ^1^H-NMR, ^13^C-NMR	[21]
72	Ferulic acid-4-O-*β*-D-glucopyranoside	C_16_H_20_O_9_	356.1107	Fruit	ESI-MS, ^1^H-NMR, ^13^C-NMR	[21]
73	Rhodioloside	C_14_H_20_O_7_	300.1209	Leaves	ESI-MS, ^1^H-NMR, ^13^C-NMR	[21]
74	5-O-Caffeoyl quinic acid methyl ester	C_17_H_20_O_9_	368.1107	Fruit	ESI-MS, ^1^H-NMR, ^13^C-NMR	[21]
75	5-O-Caffeoyl quinic acid butyl ester	C_20_H_26_O_9_	410.1577	Fruit	ESI-MS, ^1^H-NMR, ^13^C-NMR	[21]
76	5-O-Feruloyl quinic acid methyl ester	C_18_H_22_O_9_	382.1264	Fruit	ESI-MS, ^1^H-NMR, ^13^C-NMR	[21]
77	5-O-Coumaroyl quinic acid methyl ester	C_16_H_20_O_8_	340.1158	Fruit	ESI-MS, ^1^H-NMR, ^13^C-NMR	[21]

UV: ultraviolet spectrophotometry; IR: infrared spectroscopy; ^13^C-NMR: carbon-13 nuclear magnetic resonance spectrometry; ^1^H-NMR: hydrogen-1 nuclear magnetic resonance spectrometry; ESI-MS: electrospray ionization-mass spectrometry; EI-MS: electron ionization-mass spectrometry; HMBC: ^1^H-detected heteronuclear multiple bond correlation; NOESY: nuclear Overhauser effect spectroscopy; HRESI-TOF-MS: high-resolution electrospray ionization-time of flight-mass spectrometry; HPLC-MS/MS: high-performance liquid chromatography-mass spectrometry/mass spectrometry; HPLC-DAD-ESI-MS: high-performance liquid chromatography-diode array detection-electrospray ionization-mass spectrometry.

**Table 4:** Phenylpropanoid and lignin compounds isolated from *Actinidia arguta*.

No.	Name	Formula	Exact Theoretical Molecular Weight	Source	Characterization Method	Refs.
78	Chlorogenic acid	C_16_H_18_O_9_	354.0951	Leaves	HPLC	[34]
79	Quinic acid	C_7_H_12_O_6_	192.0634	Fruit	HPLC-DAD-MS/MS	[35]
80	Caffeic acid	C_9_H_8_O_4_	180.0423	Roots, leaves	EI-MS, ^1^H-NMR, ^13^C-NMR	[29]
81	trans-4-Hydroxycinnamic acid	C_9_H_8_O_3_	164.0473	Roots, leaves	EI-MS, ^1^H-NMR	[29]
82	Epipinoresinol	C_20_H_22_O_6_	358.1416	Roots	HPLC-DAD-ESI-MS	[18]
83	Pinoresinol	C_20_H_22_O_6_	358.1416	Roots, leaves	TLC, ESI-MS, ^1^H-NMR, ^13^C-NMR	[25,29]
84	Argutoside A	C_24_H_24_O_11_	488.1319	Leaves	IR, HRESI-TOF-MS, ^1^H-NMR, ^13^C-NMR, HMBC	[29]
85	Argutoside B	C_23_H_26_O_11_	465.1526	Leaves	IR, HRESI-TOF-MS, ^1^H-NMR, ^13^C-NMR, HMBC	[29]
86	Argutoside C	C_23_H_26_O_11_	465.1526	Leaves	IR, ESI-MS, HRESI-TOF-MS, ^1^H-NMR, ^13^C-NMR, HMBC	[29]
87	Argutoside D	C_23_H_26_O_11_	478.1475	Leaves	IR, HRESI-TOF-MS, ^1^H-NMR, ^13^C-NMR, HMBC, HSQC	[29]
88	(−)-Rhodolatouchol	C_10_H_14_O_3_	182.0943	Leaves	IR, HRESI-TOF-MS, ^1^H-NMR, ^13^C-NMR, HMBC	[29]
89	*p*-*E*-Coumaric acid-9-O-glucopyranoside	C_15_H_18_O_8_	326.1002	Leaves	IR, HRESI-TOF-MS, ^1^H-NMR, ^13^C-NMR, HMBC	[29]
90	*E*-Ferulic acid	C_10_H_10_O_4_	194.0579	Leaves	IR, HRESI-TOF-MS, ^1^H-NMR, ^13^C-NMR, HMBC	[29]
91	3,5-Dimethoxy-4-hydroxycinnamic alcohol	C_12_H_16_O_4_	224.1049	Leaves	IR, HRESI-TOF-MS, ^1^H-NMR, ^13^C-NMR, HMBC	[29]
92	7*S*,8*R*-Cedrusin	C_20_H_24_O_5_	344.1624	Leaves	IR, HRESI-TOF-MS, ^1^H-NMR, ^13^C-NMR, HMBC	[29]
93	Dehydroconiferyl alcohol	C_21_H_26_O_5_	358.1780	Leaves	IR, HRESI-TOF-MS, ^1^H-NMR, ^13^C-NMR, HMBC	[29]
94	(7*S*,8*S*)-3-Methoxy-3′,7-epoxy-8,4′-oxyneoligna-4,9,9′-triol	C_19_H_22_O_6_	346.1416	Leaves	IR, HRESI-TOF-MS, ^1^H-NMR, ^13^C-NMR, HMBC	[29]
95	Pinoresinol 4-O-*β*-glucopyranoside	C_26_H_32_O_11_	520.1945	Leaves	IR, HRESI-TOF-MS, ^1^H-NMR, ^13^C-NMR, HMBC	[29]
96	Alutaceuol	C_30_H_36_O_11_	572.2258	Leaves	IR, HRESI-TOF-MS, ^1^H-NMR, ^13^C-NMR, HMBC	[29]
97	Alutaceuol isomer	C_30_H_36_O_11_	572.2258	Leaves	IR, HRESI-TOF-MS, ^1^H-NMR, ^13^C-NMR, HMBC	[29]
98	(−)-(2*R*,3*R*)-Secoisolariciresinol	C_20_H_26_O_6_	362.1729	Leaves	IR, HRESI-TOF-MS, ^1^H-NMR, ^13^C-NMR, HMBC	[29]
99	Glehlinoside F	C_35_H_42_O_14_	686.2575	Leaves	IR, HRESI-TOF-MS, ^1^H-NMR, ^13^C-NMR, HMBC	[29]

HPLC: high-performance liquid chromatography; IR: infrared spectroscopy; ^13^C-NMR: carbon-13 nuclear magnetic resonance spectrometry; ^1^H-NMR: hydrogen-1 nuclear magnetic resonance spectrometry; TLC: thin-layer chromatography; HMBC: ^1^H-detected heteronuclear multiple bond correlation; ESI-MS: electrospray ionization-mass spectrometry; EI-MS: electron ionization-mass spectrometry; HSQC: heteronuclear singular quantum correlation; HRESI-TOF-MS: high-resolution electrospray ionization-time of flight-mass spectrometry; HPLC-DAD-ESI-MS: high-performance liquid chromatography-diode array detection-electrospray ionization mass spectrometry.

**Table 5:** Organic acids (esters) isolated from *Actinidia arguta*.

No.	Name	Formula	Exact Theoretical Molecular Weight	Source	Characterization Method	Refs.
100	Argutinoside A	C_20_H_24_O_12_	456.1238	Fruit	HPLC	[36]
101	Argutinoside B	C_21_H_26_O_12_	470.1424	Fruit	HPLC-DAD-MS/MS	[36]
102	Argutinoside C	C_20_H_24_O_11_	440.1319	Fruit	EI-MS, ^1^H-NMR, ^13^C-NMR	[36]
103	Argutinoside D	C_21_H_26_O_11_	454.1475	Fruit	EI-MS, ^1^H-NMR	[36]
104	Argutinoside E	C_21_H_26_O_11_	454.1475	Fruit	HPLC-DAD-ESI-MS	[36]
105	Argutinoside F	C_21_H_26_O_12_	470.1424	Fruit	TLC, ESI-MS, ^1^H-NMR, ^13^C-NMR	[36]
106	Argutinoside G	C_21_H_26_O_12_	470.1424	Fruit	IR, HRESI-TOF-MS, ^1^H-NMR, ^13^C-NMR, HMBC	[36]
107	Argutinoside H	C_22_H_28_O_12_	484.1581	Fruit	IR, HRESI-TOF-MS, ^1^H-NMR, ^13^C-NMR, HMBC	[36]
108	Argutinoside I	C_21_H_26_O_12_	470.1424	Fruit	IR, ESI-MS, HRESI-TOF-MS, ^1^H-NMR, ^13^C-NMR, HMBC	[36]
109	Butyl 2-hydroxysuccinate	C_8_H_14_O_5_	190.0841	Fruit	IR, HRESI-TOF-MS, ^1^H-NMR, ^13^C-NMR, HMBC, HSQC	[36]
110	3-O-*trans*-*p*-Coumaroyl quinic acid methyl ester	C_18_H_22_O_8_	366.1315	Fruit	IR, HRESI-TOF-MS, ^1^H-NMR, ^13^C-NMR, HMBC	[36]
111	3-O-*cis*-*p*-Coumaroyl quinic acid methyl ester	C_18_H_22_O_8_	366.1315	Fruit	IR, HRESI-TOF-MS, ^1^H-NMR, ^13^C-NMR, HMBC	[36]
112	3-O-Trans-*p*-Caffeoyl quinic acid methylester	C_18_H_22_O_9_	382.1264	Fruit	IR, HRESI-TOF-MS, ^1^H-NMR, ^13^C-NMR, HMBC	[36]
113	5-O-*trans*-*p*-Caffeoyl quinic acid methyl ester	C_18_H_22_O_8_	366.1315	Fruit	IR, HRESI-TOF-MS, ^1^H-NMR, ^13^C-NMR, HMBC	[36]
114	5-O-*cis*-*p*-Caffeoyl quinic acid methyl ester	C_18_H_22_O_8_	366.1315	Fruit	IR, HRESI-TOF-MS, ^1^H-NMR, ^13^C-NMR, HMBC	[36]
115	5-O-*trans*-*p*-Coumaroyl quinic acid methyl ester	C_18_H_22_O_9_	382.1264	Fruit	IR, HRESI-TOF-MS, ^1^H-NMR, ^13^C-NMR, HMBC	[36]
116	5-O-*cis*-*p*-Coumaroyl quinic acid methyl ester	C_18_H_22_O_9_	382.1264	Fruit	IR, HRESI-TOF-MS, ^1^H-NMR, ^13^C-NMR, HMBC	[36]
117	3-O-*trans*-*p*-Caffeoyl quinic acid butyl ester	C_21_H_28_O_9_	424.1733	Fruit	IR, HRESI-TOF-MS, ^1^H-NMR, ^13^C-NMR, HMBC	[36]
118	4-O-*trans*-*p*-Caffeoyl quinic acid butyl ester	C_21_H_28_O_9_	424.1733	Fruit	IR, HRESI-TOF-MS, ^1^H-NMR, ^13^C-NMR, HMBC	[36]
119	5-O-*trans*-*p*-Caffeoyl quinic acid butyl ester	C_21_H_28_O_8_	408.1784	Fruit	IR, HRESI-TOF-MS, ^1^H-NMR, ^13^C-NMR, HMBC	[36]
120	5-O-trans-*p*-Coumaroyl quinic acid butyl ester	C_21_H_28_O_9_	424.1733	Fruit	IR, HRESI-TOF-MS, ^1^H-NMR, ^13^C-NMR, HMBC	[36]
121	4-O-*trans*-*p*-Coumaroyl shikimic acid	C_17_H_20_O_7_	336.1209	Fruit	IR, HRESI-TOF-MS, ^1^H-NMR, ^13^C-NMR, HMBC	[36]
122	3-O-*cis*-*p*-Coumaroyl shikimic acid	C_17_H_20_O_7_	336.1209	Fruit	HPLC	[36]
123	1-Methyl-5-ethyl citrate	C_9_H_14_O_7_	234.0740	Fruit	HPLC-DAD-MS/MS	[36]
124	1,6-Dimethyl citrate	C_8_H_12_O_7_	220.0583	Fruit	EI-MS, ^1^H-NMR, ^13^C-NMR	[36]
125	1,5,6-Trimethyl citrate	C_9_H_14_O_7_	234.0740	Fruit	EI-MS, ^1^H-NMR	[36]
126	1,6-Dimethyl-5-ethyl citrate	C_10_H_16_O_7_	248.0896	Fruit	HPLC-DAD-ESI-MS	[36]
127	5-Butyl citrate	C_10_H_16_O_7_	248.0896	Fruit	TLC, ESI-MS, ^1^H-NMR, ^13^C-NMR	[36]
128	1-Methyl-6-butyl citrate	C_11_H_18_O_7_	262.1053	Fruit	IR, HRESI-TOF-MS, ^1^H-NMR, ^13^C-NMR, HMBC	[36]
129	Succinic acid	C_20_H_24_O_12_	118.0266	Leaves	IR, HRESI-TOF-MS, ^1^H-NMR, ^13^C-NMR, HMBC	[14]
130	*γ*-Quinide	C_7_H_10_O_5_	174.0528	Roots	IR, ESI-MS, HRESI-TOF-MS, ^1^H-NMR, ^13^C-NMR, HMBC	[18]
131	Octeyl-10-undecylenate	C_19_H_36_O_2_	296.2715	Stems	IR, HRESI-TOF-MS, ^1^H-NMR, ^13^C-NMR, HMBC, HSQC	[38]
132	Palmitoleic acid	C_16_H_30_O_2_	254.2246	Sprouts	IR, HRESI-TOF-MS, ^1^H-NMR, ^13^C-NMR, HMBC	[39]
133	Stearic acid	C_18_H_36_O_2_	284.2715	Sprouts	IR, HRESI-TOF-MS, ^1^H-NMR, ^13^C-NMR, HMBC	[39]
134	Oleic acid	C_18_H_34_O_2_	282.2559	Sprouts	IR, HRESI-TOF-MS, ^1^H-NMR, ^13^C-NMR, HMBC	[39]
135	*α*-Linoleic acid	C_18_H_32_O_2_	280.2402	Fruit, sprouts	IR, HRESI-TOF-MS, ^1^H-NMR, ^13^C-NMR, HMBC	[37,39]
136	*α*-Linolenic acid	C_18_H_30_O_2_	278.2246	Fruit, sprouts	IR, HRESI-TOF-MS, ^1^H-NMR, ^13^C-NMR, HMBC	[37,39]
137	Eicosadienoic acid	C_20_H_36_O_2_	308.2715	Sprouts	IR, HRESI-TOF-MS, ^1^H-NMR, ^13^C-NMR, HMBC	[39]
138	Ethyl stearate	C_19_H_37_O_2_	297.2794	Fruit	IR, HRESI-TOF-MS, ^1^H-NMR, ^13^C-NMR, HMBC	[37]

IR: infrared spectroscopy; UV: ultraviolet spectrophotometry; ^13^C-NMR: carbon-13 nuclear magnetic resonance spectrometry; ^1^H-NMR: hydrogen-1 nuclear magnetic resonance spectrometry; ESI-MS: electrospray ionization-mass spectrometry; EI-MS: electron ionization-mass spectrometry; HMBC: ^1^H-detected heteronuclear multiple bond correlation; HSQC: heteronuclear singular quantum correlation; HRESI-TOF-MS: high-resolution electrospray ionization-time of flight-mass spectrometer; HPLC-DAD-ESI-MS: high-performance liquid chromatography-diode array detection-electrospray ionization-mass spectrometry; GC: gas chromatography.

**Table 6:** Volatile components isolated from *Actinidia arguta*.

No.	Name	Formula	Exact Theoretical Molecular Weight	Source	Characterization Method	Refs.
139	*m*-Xylene	C_8_H_10_	106.0783	Roots	GC-MS	[50]
140	Naphthalene	C_10_H_8_	128.0626	Roots, flowers	GC-MS	[41,50]
141	*n*-Undecane	C_11_H_24_	156.1878	Roots	GC-MS	[50]
142	*n*-Dodecane	C_12_H_26_	170.2035	Roots, fruit	GC-MS	[41,50]
143	*n*-Tetradecane	C_14_H_30_	198.2348	Roots, fruit	GC-MS	[41,50]
144	*n*-Heptadecane	C_17_H_36_	240.2817	Roots, flowers, fruit	GC-MS	[41,46,50]
145	*n*-Eicosane	C_20_H_42_	282.3287	Roots, flowers	GC-MS	[41,46,50]
146	2,6,10-Trimethyldodecane	C_15_H_32_	212.2504	Roots	GC-MS	[50]
147	2,6,10,14-Tetramethylpentadecane	C_19_H_40_	268.3130	Roots	GC-MS	[50]
148	8-Methylheptadecane	C_18_H_38_	254.2974	Roots	GC-MS	[50]
149	Ethylmethylundecanol	C_14_H_30_O	214.2297	Roots	GC-MS	[50]
150	Methyl pentadecanoate	C_16_H_32_O_2_	256.2402	Roots	GC-MS	[50]
151	Dibutyl phthalate	C_16_H_22_O_4_	278.1518	Roots	GC-MS	[50]
152	2,4,6-Trimethyl decanoic acid	C_13_H_26_O_2_	214.1933	Roots	GC-MS	[50]
153	3,7-Dimethyl-1,8-Nonadiene	C_11_H_20_	152.1565	Roots	GC-MS	[50]
154	(3*E*)-3-Undecene	C_11_H_22_	154.1722	Roots	GC-MS	[50]
155	1,6-Nonadien-3-ol,3,7-dimethyl-,acetate	C_13_H_22_O_2_	210.1620	Roots	GC-MS	[50]
156	Ethyl acetate	C_4_H_8_O_2_	88.0524	Flowers, fruit	GC-MS	[41,43]
157	Butanoic acid, methyl ester	C_5_H_10_O_2_	102.0681	Fruit	GC-MS	[43]
158	Pyridine	C_5_H_5_N	79.0422	Fruit	GC-MS	[43]
159	(*E*)-2-Hexenal	C_6_H_10_O	98.0732	Fruit	GC-MS	[43]
160	1-Hexanol	C_6_H_14_O	102.1045	Flowers, fruit	GC-MS	[41,42,43,46]
161	Hexanoic acid, ethyl ester	C_8_H_16_O_2_	144.1150	Fruit	GC-MS	[43]
162	3-Cyclohexen-1-ol,4-methyl-1-(methylethyl)	C_10_H_18_O	154.1358	Fruit	GC-MS	[43]
163	Ethyl butyrate	C_6_H_12_O_2_	116.0837	Fruit	GC-MS	[42,46]
164	2-Furaldehyde	C_5_H_4_O_2_	96.0211	Fruit	GC-MS	[42]
165	2-Hexenal	C_6_H_10_O	98.0732	Fruit	GC-MS	[42,46]
166	(*E*)-3-Hexen-1-ol	C_6_H_12_O	100.0888	Fruit	GC-MS	[42]
167	*cis*-Hex-2-en-1-ol	C_6_H_12_O	100.0888	Fruit	GC-MS	[42]
168	2-Hexen-1-ol	C_6_H_12_O	100.0888	Fruit	GC-MS	[42,43]
169	Dihydrofuran-2(3H)-one	C_4_H_6_O_2_	86.0386	Fruit	GC-MS	[42]
170	(1*S*)-(−)-*α*-Pinene	C_10_H_16_	136.1252	Fruit	GC-MS	[42]
171	Benzaldehyde	C_7_H_6_O	106.0419	Fruit	GC-MS	[42]
172	5-Methylfurfural	C_6_H_6_O_2_	110.0368	Fruit	GC-MS	[42]
173	1-Octen-3-ol	C_8_H_16_O	128.1201	Fruit	GC-MS	[42]
174	Benzene,1-methyl-2-(1-methylethyl)-	C_10_H_14_	134.1096	Fruit	GC-MS	[42]
175	1-Methyl-4-methyl ethenyl cyclohexene	C_10_H_16_	136.1252	Fruit	GC-MS	[42]
176	1,3,3-Trimethyl-2-oxabicyclo[2.2.2]octane	C_10_H_18_O	154.1358	Fruit	GC-MS	[42]
177	Benzyl alcohol	C_7_H_8_O	108.0575	Flowers, fruit	GC-MS	[42]
178	*o*-Cresol	C_7_H_8_O	108.0575	Fruit	GC-MS	[42]
179	Phenylacetaldehyde	C_8_H_8_O	120.0575	Fruit	GC-MS	[42]
180	4-Isopropyl-1-methyl-1,4-cyclohexadiene	C_10_H_16_	136.1252	Fruit	GC-MS	[42]
181	*α*-Terpinolene	C_10_H_16_	136.1252	Flowers, fruit	GC-MS	[41,42,45]
182	1-Methyl-4-(prop-1-en-2-yl)benzene	C_10_H_12_	132.0939	Fruit	GC-MS	[42]
183	Methyl benzoate	C_8_H_8_O_2_	136.0524	Fruit	GC-MS	[41,42]
184	1,6-Octadien-3-ol,3,7-dimethyl	C_10_H_18_O	154.1358	Fruit	GC-MS	[42]
185	Ethyl benzoate	C_9_H_10_O_2_	150.0681	Fruit	GC-MS	[41,42]
186	Terpinen-4-ol	C_10_H_18_O	154.1358	Fruit	GC-MS	[42]
187	Trimethylbenzene	C_9_H_12_	120.0939	Flowers, fruit	GC-MS	[41,42]
188	*α*-Terpineol	C_10_H_18_O	154.1358	Fruit	GC-MS	[42,46]
189	4-(2,6,6-Trimethylcyclohex-2-en-1-yl)but-3-en-2-one	C_13_H_20_O	192.1514	Fruit	GC-MS	[42]
190	N,N-Dibutylformamide	C_9_H_19_NO	157.1467	Fruit	GC-MS	[42]
191	2-Methoxy-4-vinylphenol	C_9_H_10_O_2_	150.0681	Fruit	GC-MS	[42]
192	2-(Benzo[d][1,3]dioxol-5-yl)-6-chloroimidazo[1,2-b]pyridazine	C_13_H_8_N_3_O_2_Cl	273.0305	Fruit	GC-MS	[42]
193	Triethyl citrate	C_12_H_20_O_7_	276.1209	Fruit	GC-MS	[42]
194	Methyl tetradecanoate	C_15_H_30_O_2_	242.2246	Fruit	GC-MS	[42]
195	Myristic acid	C_14_H_28_O_2_	228.2089	Fruit	GC-MS	[42]
196	1,2-Benzene-3,4,5,6-d_4_-dicarboxylicacid, bis(2-methylpropyl) ester	C_16_H_22_O_4_	282.1769	Fruit	GC-MS	[42]
197	1-(2,4-Difluorophenyl)piperazine	C_10_H_12_F_2_N_2_	198.0969	Fruit	GC-MS	[42]
198	Ethyl palmitate	C_18_H_36_O_2_	284.2715	Fruit	GC-MS	[42]
199	Methyl linolenate	C_19_H_32_O_2_	292.2402	Fruit	GC-MS	[41,42]
200	Ethyl linoleate	C_20_H_36_O_2_	308.2715	Fruit	GC-MS	[41,42]
201	Ethyl linolenate	C_20_H_34_O_2_	306.2559	Fruit	GC-MS	[41,42]
202	Ethanol	C_2_H_6_O	46.0619	Fruit	GC-MS	[44]
203	*α*-Pinene	C_10_H_16_	136.1252	Flowers, fruit	GC-MS	[41,44]
204	*β*-Pinene	C_10_H_16_	136.1252	Flowers, fruit	GC-MS	[41,44]
205	*β*-Myrcene	C_10_H_16_	136.1252	Flowers, fruit	GC-MS	[41,44]
206	Benzene,1-methyl-3-(1-methylethyl)-	C_10_H_14_	134.1096	Fruit	GC-MS	[44]
207	Dipentene	C_10_H_16_	136.1252	Fruit	GC-MS	[44]
208	(1*R*-*trans*) 1-Methyl-4-(1-methylethenyl)-2-cyclohexene-1-ol	C_10_H_16_O	152.1201	Fruit	GC-MS	[44]
209	4,6,6-Trimethyl-bicyclo[3.1.1]hept-3-en-2-one	C_10_H_14_O	150.1045	Fruit	GC-MS	[44]
210	9,12-Octadecadienoic acid	C_18_H_32_O_2_	280.2402	Fruit, seeds	GC-MS	[47,48]
211	Palmitic acid	C_16_H_32_O_2_	256.2402	Fruit, seeds	GC-MS	[48]
212	Linolenic acid	C_18_H_30_O_2_	278.2246	Fruit, seeds	GC-MS	[48]
213	Erucic acid	C_22_H_42_O_2_	338.3815	Seeds	GC-MS	[48]
214	Gondoic acid	C_20_H_38_O_2_	310.2872	Seeds	GC-MS	[48]
215	(10*Z*,13*Z*)-Octadeca-10,13-dienoic acid	C_18_H_32_O_2_	280.2402	Fruit	GC-MS	[47]
216	Eicosanoic-12,12,13,13-d_4_-acid	C_20_H_40_O_2_	316.3279	Fruit	GC-MS	[47]
217	9-Octadecenoic acid	C_18_H_34_O_2_	282.2559	Seeds	GC-MS	[49]
218	Eucalyptol	C_10_H_18_O	154.1358	Flowers, fruit	GC-MS	[41,44]
219	1-Methyl-4-(1-methylethenyl)-cyclohexene	C_10_H_16_	136.1252	Fruit	GC-MS	[44]
220	3,4-Dimethylbicyclo[3.2.1]oct-2-ene	C_10_H_16_	136.1252	Fruit	GC-MS	[44]
221	Methyl acetate	C_3_H_6_O_2_	74.0368	Fruit	GC-MS	[41,45]
222	Acetic acid	C_2_H_4_O_2_	60.0211	Flowers, fruit	GC-MS	[41,45]
223	1,3,5,7-Cyclooctatetraene	C_8_H_8_	104.0626	Fruit	GC-MS	[45]
224	Styrene	C_8_H_8_	104.0626	Fruit	GC-MS	[41,45]
225	2-Methyl-bicyclo[3.1.0]hexan-2-en	C_7_H_10_	94.0783	Fruit	GC-MS	[45]
226	*p*-Cymene	C_10_H_14_	134.1096	Fruit	GC-MS	[41,45]
227	3-Carene	C_10_H_16_	136.1252	Fruit	GC-MS	[45]
228	4-Isopropyl-1-methyl-1,4-cyclohexadiene	C_10_H_16_	136.1252	Fruit	GC-MS	[45]
229	Methyl heptenone	C_8_H_14_O	126.1045	Fruit	GC-MS	[45]
230	1-Ethyl-3,5-dimethylbenzene	C_10_H_14_	134.1096	Fruit	GC-MS	[45]
231	Camphor	C_10_H_16_O	152.1201	Flowers, fruit	GC-MS	[41]
232	*β*-Caryophyllene	C_15_H_24_	204.1878	Flowers	GC-MS	[41]
233	2,6-Dimethyl-6-hydroxyocta-2,7-dienal	C_10_H_16_O_2_	168.1150	Flowers	GC-MS	[41]
234	2,6-Dimethylocta-3,7-diene-2,6-diol	C_10_H_18_O_2_	170.1307	Flowers	GC-MS	[41]
235	*E*,*E*-*α*-Farnesene	C_15_H_24_	204.1878	Flowers	GC-MS	[41]
236	*Z*,*E*-Farnesol	C_15_H_26_O	222.1984	Flowers	GC-MS	[41]
237	*E*,*E*-Farnesyl acetate	C_17_H_28_O_2_	264.2089	Flowers	GC-MS	[41]
238	Geranylacetone	C_13_H_22_O	194.1671	Flowers	GC-MS	[41]
239	Germacrene D	C_15_H_28_O	208.2191	Flowers	GC-MS	[41]
240	Hexahydrofarnesylacetone	C_18_H_36_O	268.2766	Flowers	GC-MS	[41]
241	*E*-8-Hydroxylinalool	C_10_H_18_O_2_	170.1307	Flowers	GC-MS	[41]
242	*Z*-8-Hydroxylinalool	C_10_H_18_O_2_	170.1307	Flowers	GC-MS	[41]
243	Lilac alcohol a	C_10_H_18_O_2_	170.1307	Flowers	GC-MS	[41]
244	Lilac alcohol b	C_10_H_18_O_2_	170.1307	Flowers	GC-MS	[41]
245	Lilac alcohol c	C_10_H_18_O_2_	170.1307	Flowers	GC-MS	[41]
246	Lilac alcohol d	C_10_H_18_O_2_	170.1307	Flowers	GC-MS	[41]
247	Lilac aldehyde 1	C_10_H_16_O_2_	168.1150	Flowers	GC-MS	[41]
248	Lilac aldehyde 2	C_10_H_16_O_2_	168.1150	Flowers	GC-MS	[41]
249	Lilac aldehyde 3	C_10_H_16_O_2_	168.1150	Flowers	GC-MS	[41]
250	Lilac aldehyde 4	C_10_H_16_O_2_	168.1150	Flowers	GC-MS	[41]
251	Limonene	C_10_H_16_	136.1252	Flowers, fruit	GC-MS	[41]
252	Linalool	C_10_H_18_O	154.1358	Flowers, fruit	GC-MS	[41]
253	*cis*-Linalool oxide	C_10_H_18_O_2_	170.1307	Flowers	GC-MS	[41]
254	*trans*-Linalool oxide	C_10_H_18_O_2_	170.1307	Flowers	GC-MS	[41]
255	6-Methylhept-5-en-2-one	C_8_H_14_O	126.1045	Flowers, fruit	GC-MS	[41]
256	Ocimene	C_10_H_16_	136.1252	Flowers	GC-MS	[41]
257	*E*-*β*-Ocimene	C_10_H_16_	136.1252	Flowers, fruit	GC-MS	[41]
258	Phytol	C_20_H_40_O	296.3079	Flowers	GC-MS	[41]
259	Squalene	C_30_H_50_	410.3913	Flowers, fruit	GC-MS	[41,46]
260	3,7,11,15-Tetramethyl hexadeca-6,10,14-trienol	C_20_H_36_O	292.2766	Flowers	GC-MS	[41]
261	Camphene	C_10_H_16_	136.1252	Fruit	GC-MS	[41]
262	2-Carene	C_10_H_16_	136.1252	Fruit	GC-MS	[41]
263	*cis*-Carveol	C_10_H_16_O	152.1201	Fruit	GC-MS	[41]
264	Carvone	C_10_H_14_O	150.1045	Fruit	GC-MS	[41]
265	*Endo*-5,5,6-trimethylnorbornan-2-one	C_10_H_16_O	152.1201	Fruit	GC-MS	[41]
266	*p*-Mentha-1,3,8-triene	C_10_H_14_	134.1096	Fruit	GC-MS	[41]
267	*p*-Menth-1-en-4-ol	C_10_H_18_O	154.1358	Fruit	GC-MS	[41]
268	Menthol	C_10_H_20_O	156.1514	Fruit	GC-MS	[41]
269	1-Methyl-4-(1-methylethenyl)benzene	C_10_H_12_	132.0939	Fruit	GC-MS	[41]
270	1-Methyl-4-(1-methylethyl)-cyclohex-2-enol	C_10_H_18_O	154.1358	Fruit	GC-MS	[41]
271	*Z*-*β*-Ocimene	C_10_H_16_	136.1252	Fruit	GC-MS	[41]
272	*β*-Phellandrene	C_10_H_16_	136.1252	Fruit	GC-MS	[41]
273	Sabinene	C_10_H_16_	136.1252	Fruit	GC-MS	[41]
274	*α*-Terpinene	C_10_H_16_	136.1252	Fruit	GC-MS	[41]
275	*β*-Terpinene	C_10_H_16_	136.1252	Fruit	GC-MS	[41]
276	*δ*-Terpinene	C_10_H_16_	136.1252	Fruit	GC-MS	[41]
277	*α*-Terpineol	C_10_H_18_O	154.1358	Fruit	GC-MS	[41]
278	Ethylbenzaldehyde	C_9_H_10_O	134.0732	Flowers	GC-MS	[41]
279	Benzene	C_6_H_6_	78.0470	Flowers	GC-MS	[41]
280	Benzyl benzoate	C_14_H_12_O_2_	212.0837	Flowers, fruit	GC-MS	[41]
281	Ethylbenzaldehyde	C_9_H_10_O	134.0732	Flowers, fruit	GC-MS	[41]
282	2-(4-Hydroxyphenyl)ethanol	C_8_H_10_O_2_	138.0681	Flowers	GC-MS	[41]
283	Methoxybenzene	C_8_H_10_O	122.0732	Flowers	GC-MS	[41]
284	2-(4-Methoxyphenyl)ethanol	C_9_H_12_O_2_	152.0837	Flowers, fruit	GC-MS	[41]
285	Methyl 4-Methoxybenzoate	C_9_H_10_O_3_	166.0630	Flowers	GC-MS	[41]
286	Methyl salicylate	C_8_H_8_O_3_	152.0473	Flowers	GC-MS	[41]
287	Phenol	C_6_H_6_O	94.0619	Flowers	GC-MS	[41]
288	2-Phenylethanal	C_8_H_8_O	120.0575	Flowers	GC-MS	[41]
289	2-Phenylethanol	C_8_H_10_O	122.0732	Flowers	GC-MS	[41]
290	2-Phenylethyl acetate	C_10_H_12_O_2_	164.0837	Flowers	GC-MS	[41]
291	Dimethylbenzaldehyde	C_9_H_10_O	134.0732	Fruit	GC-MS	[41]
292	1,2-Dimethylbenzene	C_8_H_10_	106.0783	Fruit	GC-MS	[41]
293	Hex-3(*Z*)-enyl acetate	C_8_H_14_O_2_	142.0994	Flowers	GC-MS	[41]
294	3-Methylbutyl acetate	C_7_H_14_O_2_	130.0994	Flowers	GC-MS	[41]
295	Butyl acetate	C_6_H_12_O_2_	116.0837	Fruit	GC-MS	[41]
296	Dimethyl carbonate	C_3_H_6_O_3_	90.0317	Fruit	GC-MS	[41]
297	Ethyl (2*E*)-2-butenoate	C_6_H_10_O_2_	114.0681	Fruit	GC-MS	[41]
298	Ethyl butanoate	C_6_H_12_O_2_	116.0837	Fruit	GC-MS	[41]
299	Ethyl decanoate	C_12_H_24_O_2_	200.1776	Fruit	GC-MS	[41]
300	Ethyl heptanoate	C_9_H_18_O_2_	158.1307	Fruit	GC-MS	[41]
301	Ethyl hexadecanoate	C_18_H_36_O_2_	284.2715	Fruit	GC-MS	[41]
302	Ethyl hexadec-9-enoate	C_18_H_34_O_2_	282.2559	Fruit	GC-MS	[41]
303	Ethyl hexanoate	C_8_H_16_O_2_	144.1150	Flowers, fruit	GC-MS	[41]
304	Ethyl hexa-2,4-dienoate	C_8_H_12_O_2_	140.0837	Fruit	GC-MS	[41]
305	Ethyl hex-2-enoate	C_8_H_14_O_2_	142.0994	Fruit	GC-MS	[41]
306	Ethyl hex-3-enoate	C_8_H_14_O_2_	142.0994	Fruit	GC-MS	[41]
307	Ethyl 2-methylbutanoate	C_7_H_14_O_2_	130.0994	Fruit	GC-MS	[41]
308	Ethyl 3-methylbutanoate	C_7_H_14_O_2_	130.0994	Fruit	GC-MS	[41]
309	Ethyl 2-methylpropanoate	C_6_H_12_O_2_	116.0837	Fruit	GC-MS	[41]
310	Ethyl octanoate	C_10_H_20_O_2_	172.1463	Fruit	GC-MS	[41]
311	Ethyl (4*Z*)-oct-4-enoate	C_10_H_18_O_2_	170.1307	Fruit	GC-MS	[41]
312	Ethyl oleate	C_20_H_38_O_2_	310.2872	Fruit	GC-MS	[41]
313	Ethyl pentanoate	C_7_H_14_O_2_	130.0994	Fruit	GC-MS	[41]
314	Ethyl propanoate	C_5_H_10_O_2_	102.0681	Fruit	GC-MS	[41]
315	Hexadecyl acetate	C_18_H_36_O_2_	284.2715	Fruit	GC-MS	[41]
316	Methyl butanoate	C_5_H_10_O_2_	102.0681	Fruit	GC-MS	[41]
317	1-Methylethyl tetradecanoate	C_17_H_34_O_2_	270.2559	Fruit	GC-MS	[41]
318	Methyl hexadecanoate	C_17_H_34_O_2_	270.2559	Fruit	GC-MS	[41]
319	Methyl linoleate	C_19_H_34_O_2_	294.2559	Fruit	GC-MS	[41]
320	Methyl octadecanoate	C_19_H_38_O_2_	298.2872	Fruit	GC-MS	[41]
321	Methyl oleate	C_19_H_36_O_2_	296.2715	Fruit	GC-MS	[41]
322	Methyl prop-2-enoate	C_4_H_6_O_2_	86.0368	Fruit	GC-MS	[41]
323	Propyl butanoate	C_7_H_14_O_2_	130.0994	Fruit	GC-MS	[41]
324	2-Methylbutanal	C_5_H_10_O	86.0732	Flowers	GC-MS	[41]
325	3-Methylbut-2-enal	C_5_H_8_O	84.0575	Flowers	GC-MS	[41]
326	2-Methylpropanal	C_4_H_8_O	72.0575	Flowers	GC-MS	[41]
327	Undecanal	C_11_H_22_O	170.1671	Flowers	GC-MS	[41]
328	Acetaldehyde	C_2_H_4_O	44.0262	Flowers, fruit	GC-MS	[41]
329	Decanal	C_10_H_20_O	156.1514	Flowers, fruit	GC-MS	[41]
330	(2*E*,4*E*)-2,4-Heptadienal	C_7_H_10_O	110.0732	Fruit	GC-MS	[41]
331	Heptanal	C_7_H_14_O	114.1045	Flowers, fruit	GC-MS	[41]
332	(2*Z*)-2-Heptenal	C_7_H_12_O	112.0888	Fruit	GC-MS	[41]
333	Hexanal	C_6_H_12_O	100.0888	Flowers, fruit	GC-MS	[41,46]
334	(2*E*)-2-Hexenal	C_6_H_10_O	98.0732	Fruit	GC-MS	[41]
335	(2*Z*)-2-Hexenal	C_6_H_10_O	98.0732	Fruit	GC-MS	[41]
336	(3*E*)-3-Hexenal	C_6_H_10_O	98.0732	Fruit	GC-MS	[41]
337	(3*Z*)-3-Hexenal	C_6_H_10_O	98.0732	Fruit	GC-MS	[41]
338	3-Methylbutanal	C_5_H_10_O	86.0732	Flowers, fruit	GC-MS	[41]
339	2-Methylpentenal	C_6_H_10_O	98.0732	Fruit	GC-MS	[41]
340	(2*E*,6*Z*)-Nona-2,6-dienal	C_9_H_14_O	138.1045	Fruit	GC-MS	[41]
341	Nonanal	C_9_H_18_O	142.1358	Flowers, fruit	GC-MS	[41]
342	(2*E*)-Non-2-enal	C_9_H_16_O	140.1201	Fruit	GC-MS	[41]
343	Octanal	C_8_H_16_O	128.1201	Flowers, fruit	GC-MS	[41]
344	(2*E*)-Oct-2-enal	C_8_H_14_O	126.1045	Fruit	GC-MS	[41]
345	Propanal	C_3_H_6_O	58.0419	Fruit	GC-MS	[41]
346	Acetone	C_3_H_6_O	58.0419	Flowers, fruit	GC-MS	[41]
347	Butan-2-one	C_4_H_8_O	72.0575	Flowers, fruit	GC-MS	[41]
348	Butane-2,3-dione	C_4_H_6_O_2_	86.0368	Flowers	GC-MS	[41]
349	3-Hydroxybutan-2-one	C_4_H_8_O_2_	88.0524	Flowers, fruit	GC-MS	[41]
350	7,8-Dehydro-*β*-ionone	C_13_H_22_O	194.1671	Flowers	GC-MS	[41]
351	*β*-Ionone	C_13_H_20_O	192.1514	Flowers	GC-MS	[41]
352	Jasmone	C_11_H_16_O	164.1201	Flowers	GC-MS	[41]
353	2-Methylpentan-3-one	C_6_H_12_O	100.0888	Flowers	GC-MS	[41]
354	4-Methylpentan-2-one	C_6_H_12_O	100.0888	Flowers	GC-MS	[41]
355	Octan-3-one	C_8_H_6_O	128.1201	Flowers	GC-MS	[41]
356	Pentadecan-2-one	C_15_H_30_O	226.2297	Flowers	GC-MS	[41]
357	Cyclopentanone	C_5_H_8_O	84.0575	Fruit	GC-MS	[41]
358	4-Hydroxy-4-methylpentan-2-one	C_6_H_12_O_2_	116.0837	Fruit	GC-MS	[41]
359	4-Methylpent-3-en-2-one	C_6_H_10_O	98.0732	Fruit	GC-MS	[41]
360	Octan-2,3-dione	C_8_H_14_O_2_	142.0994	Fruit	GC-MS	[41]
361	Penten-3-one	C_5_H_8_O	84.0575	Fruit	GC-MS	[41]
362	(3*E*)-3-Penten-2-one	C_5_H_8_O	84.0575	Fruit	GC-MS	[41]
363	Butanol	C_4_H_10_O	74.0732	Flowers	GC-MS	[41]
364	Butan-2-ol	C_4_H_10_O	74.0732	Flowers	GC-MS	[41]
365	2-Ethylhexanol	C_8_H_18_O	130.1358	Flowers	GC-MS	[41]
366	Hexadecanol	C_16_H_34_O	242.2610	Flowers, fruit	GC-MS	[41]
367	Methanol	CH_4_O	32.0262	Flowers	GC-MS	[41]
368	1-Methoxypropan-2-ol	C_4_H_10_O_2_	90.0681	Flowers	GC-MS	[41]
369	2-Methylbutanol	C_5_H_12_O	88.0888	Flowers	GC-MS	[41]
370	3-Methylbutanol	C_5_H_12_O	88.0888	Flowers, fruit	GC-MS	[41]
371	3-Methylbut-2-enol	C_5_H_10_O	86.0732	Flowers	GC-MS	[41]
372	3-Methylbut-3-enol	C_5_H_10_O	86.0732	Flowers	GC-MS	[41]
373	2-Methylbut-3-en-2-ol	C_5_H_10_O	86.0732	Flowers	GC-MS	[41]
374	2-Methylpropanol	C_4_H_10_O	74.0732	Flowers, fruit	GC-MS	[41]
375	Nonanol	C_9_H_20_O	144.1514	Flowers, fruit	GC-MS	[41]
376	Pentanol	C_5_H_12_O	88.0888	Flowers, fruit	GC-MS	[41]
377	Pentan-2-ol	C_5_H_12_O	88.0888	Flowers	GC-MS	[41]
378	Pentan-3-ol	C_5_H_12_O	88.0888	Flowers	GC-MS	[41]
379	Penten-3-ol	C_5_H_10_O	86.0732	Flowers, fruit	GC-MS	[41]
380	Propanol	C_3_H_8_O	60.0575	Flowers, fruit	GC-MS	[41]
381	Octanol	C_8_H_18_O	130.1358	Flowers	GC-MS	[41]
382	Octan-4-ol	C_8_H_18_O	130.1358	Flowers	GC-MS	[41]
383	Oct-1-en-3-ol	C_8_H_16_O	128.1201	Flowers	GC-MS	[41]
384	Decanol	C_10_H_22_O	158.1671	Fruit	GC-MS	[41]
385	Dodecanol	C_12_H_26_O	186.1984	Fruit	GC-MS	[41]
386	Heptanol	C_7_H_16_O	116.1201	Fruit	GC-MS	[41]
387	(2*E*)-2-Hexen-1-ol	C_6_H_12_O	100.0888	Fruit	GC-MS	[41]
388	(2*Z*)-2-Hexen-1-ol	C_6_H_12_O	100.0888	Fruit	GC-MS	[41]
389	(3*Z*)-3-Hexen-1-ol	C_6_H_12_O	100.0888	Fruit	GC-MS	[41]
390	Octanol	C_8_H_18_O	130.1358	Fruit	GC-MS	[41]
391	Oct-1-en-3-ol	C_8_H_16_O	128.1201	Fruit	GC-MS	[41]
392	(2*E*)-2-Penten-1-ol	C_5_H_10_O	86.0732	Fruit	GC-MS	[41]
393	Dodecanoic acid	C_12_H_24_O_2_	200.1776	Flowers	GC-MS	[41]
394	Heptanoic acid	C_7_H_14_O_2_	130.0994	Flowers	GC-MS	[41]
395	Hexanoic acid	C_6_H_12_O_2_	116.0837	Flowers	GC-MS	[41]
396	3-Methylbutanoic acid	C_5_H_10_O_2_	102.0681	Flowers	GC-MS	[41]
397	Nonanoic acid	C_9_H_18_O_2_	158.1307	Flowers	GC-MS	[41]
398	Octanoic acid	C_8_H_16_O_2_	144.1150	Flowers	GC-MS	[41]
399	Butanoic acid	C_4_H_8_O_2_	88.0524	Fruit	GC-MS	[41]
400	Heptacosane	C_27_H_56_	380.4382	Flowers	GC-MS	[41,46]
401	Hexacosane	C_18_H_54_	366.4226	Flowers	GC-MS	[41,46]
402	Hexadecane	C_16_H_34_	226.2661	Flowers, fruit	GC-MS	[41]
403	Hexa-1,4-diene	C_6_H_10_	82.0783	Flowers	GC-MS	[41]
404	(2*Z*,4*Z*)-2,4-Hexadiene	C_6_H_10_	82.0783	Flowers	GC-MS	[41]
405	3-Methylcyclopentene	C_6_H_10_	82.0783	Flowers	GC-MS	[41]
406	3-Methylpenta-1,3-diene	C_6_H_10_	82.0783	Flowers	GC-MS	[41]
407	Nonacosane	C_29_H_60_	408.4695	Flowers	GC-MS	[41,46]
408	Nonadecane	C_19_H_40_	268.3130	Flowers, fruit	GC-MS	[41,46]
409	Nonane	C_9_H_20_	128.1565	Flowers	GC-MS	[41]
410	Octane	C_8_H_18_	114.1409	Flowers	GC-MS	[41]
411	Pentacosane	C_25_H_52_	352.4069	Flowers	GC-MS	[41,46]
412	Pentadecane	C_15_H_32_	212.2504	Flowers, fruit	GC-MS	[41]
413	Tricosane	C_23_H_48_	324.3756	Flowers	GC-MS	[41,46]
414	2,6-Dimethyldecane	C_12_H_26_	170.2035	Fruit	GC-MS	[41]
415	2-Methylpenta-1,3-diene	C_6_H_10_	82.0783	Fruit	GC-MS	[41]
416	2-Methoxy-2-methylpropane	C_5_H_12_O	88.0888	Fruit	GC-MS	[41]
417	Octadecane	C_18_H_38_	254.2974	Fruit	GC-MS	[41,46]
418	Tridecane	C_13_H_28_	184.2191	Fruit	GC-MS	[41]
419	Bis(1-methylethyl)disulphide	C_6_H_14_S_2_	150.0537	Flowers, fruit	GC-MS	[41]
420	Carbon disulphide	CS_2_	75.9441	Flowers	GC-MS	[41]
421	Dimethyl disulphide	C_2_H_6_S_2_	93.9911	Flowers	GC-MS	[41]
422	Butanenitrile	C_4_H_7_N	69.0578	Flowers	GC-MS	[41]
423	Methenamine	C_6_H_12_N_4_	140.1062	Flowers	GC-MS	[41]
424	2-Methylbutanenitrile	C_5_H_9_N	83.0735	Flowers	GC-MS	[41]
425	Tetrahydrofuran	C_4_H_8_O	72.0575	Flowers, fruit	GC-MS	[41]
426	Ethyl 2-furancarboxylate	C_7_H_8_O_3_	140.0473	Fruit	GC-MS	[41]
427	2-Furancarboxaldehyde	C_5_H_4_O_2_	96.0211	Fruit	GC-MS	[41]
428	4-Methoxy-2,5-dimethyl-3(2H)-furanone	C_7_H_10_O_3_	142.0630	Fruit	GC-MS	[41]
429	2-Methylfuran	C_5_H_6_O	82.0419	Fruit	GC-MS	[41]
430	5-Methyl-2-furfural	C_6_H_6_O_2_	110.0368	Fruit	GC-MS	[41]
431	Methyl 2-furoate	C_6_H_6_O_3_	126.0317	Fruit	GC-MS	[41]
432	*n*-Docosane	C_21_H_44_	296.3443	Stems	IR, EI-MS	[38,46]
433	Benzeneethanol	C_8_H_10_O	122.0732	Fruit	GC-MS	[46]
434	Benzoic acid ethyl ester	C_9_H_10_O_2_	150.0681	Fruit	GC-MS	[46]
435	1-Eicosanol	C_20_H_42_O	298.3236	Fruit	GC-MS	[46]
436	Neophytadiene	C_20_H_38_	278.2974	Fruit	GC-MS	[46]
437	Cyclotetradecane	C_14_H_28_	196.2191	Fruit	GC-MS	[46]
438	Isopropyl palmitate	C_19_H_38_O_2_	298.2872	Fruit	GC-MS	[46]
439	1-Octadecene	C_18_H_36_	252.2817	Fruit	GC-MS	[46]
440	Heneicosane	C_21_H_44_	296.3443	Fruit	GC-MS	[46]
441	Decylcyclohexane	C_16_H_32_	224.2504	Fruit	GC-MS	[46]
442	Hexadecanamide	C_16_H_33_NO	255.2562	Fruit	GC-MS	[46]
443	1-Naphthalenamine, N-phenyl-	C_16_H_13_N	219.1048	Fruit	GC-MS	[46]
444	9-Octadecenamide	C_18_H_35_NO	281.2719	Fruit	GC-MS	[46]
445	Octadecanamide	C_18_H_37_NO	283.2875	Fruit	GC-MS	[46]
446	Tetracosane	C_24_H_50_	338.3913	Fruit	GC-MS	[46]
447	Linoleic acid butyl ester	C_22_H_40_O_2_	336.3028	Fruit	GC-MS	[46]
448	Octacosane	C_28_H_58_	394.4539	Fruit	GC-MS	[46]
449	Schizandrin	C_24_H_32_O_7_	432.2148	Fruit	GC-MS	[46]
450	Octadecane	C_18_H_38_	254.2974	Fruit	GC-MS	[46]
451	Triacontane	C_30_H_62_	422.4852	Fruit	GC-MS	[46]
452	*β*-Tocopherol	C_28_H_48_O_2_	416.3654	Fruit	GC-MS	[46]
453	Hentriacontane	C_31_H_64_	436.5008	Fruit	GC-MS	[46]
454	Hexacosanol	C_26_H_54_O	382.4175	Fruit	GC-MS	[46]
455	Tritriacontane	C_33_H_68_	464.5321	Fruit	GC-MS	[46]
456	Cholest-5-en-3-ol	C_27_H_46_O	386.3549	Fruit	GC-MS	[46]
457	Campesterol	C_28_H_48_O	400.3705	Fruit	GC-MS	[46]
458	Stigmasta-5,22-dien-3-ol	C_29_H_48_O	412.3705	Fruit	GC-MS	[46]
459	*γ*-Sitosterol	C_29_H_50_O	414.3862	Fruit	GC-MS	[46]
460	*β*-Amyrin	C_30_H_50_O	426.3862	Fruit	GC-MS	[46]
461	*α*-Amyrin	C_30_H_50_O	426.3862	Fruit	GC-MS	[46]
462	Stigmast-7-en-3-ol	C_29_H_50_O	414.3862	Fruit	GC-MS	[46]
463	9,19-Cyclolanostan-3-ol,24-methylene	C_31_H_52_O	440.4018	Fruit	GC-MS	[46]
464	9,19-Cyclolanostan-3-ol,acetate	C_32_H_54_O_2_	470.4124	Fruit	GC-MS	[46]
465	D:A-Friedooleanan-3-one	C_30_H_50_O	426.3862	Fruit	GC-MS	[46]

GC-MS: Gas chromatography-mass spectrometry.

**Table 7:** Other compounds isolated from *Actinidia arguta*.

No.	Name	Formula	Exact Theoretical Molecular Weight	Source	Characterization Method	Refs.
466	*β*-Sitosterol	C_29_H_50_O	414.3862	Roots	IR, EI-MS, ^1^H-NMR	[17]
467	Daucosterol	C_35_H_60_O_6_	576.4390	Stems	TLC, IR, ^13^C-NMR	[19]
468	Ergosterol-4,6,8 (14), 22-tetraene-3-one	C_28_H_39_O_2_	407.2950	Roots	HPLC-DAD-ESI-MS	[18]
469	Aconitine	C_34_H_47_NO_11_	645.3149	Fruit	HPLC-MS	[51]
470	Berberine	C_20_H_18_NO_4_	336.1230	Fruit	HPLC-MS	[51]
471	Corydaline	C_34_H_47_NO_11_	369.1940	Fruit	HPLC-MS	[51]
472	Tetrahydropalmatine	C_34_H_47_NO_11_	355.1784	Fruit	HPLC-MS	[51]
473	Hypaconitine	C_33_H_45_NO_10_	615.3043	Fruit	HPLC-MS	[51]
474	Physostigmine	C_34_H_47_NO_11_	275.1634	Fruit	HPLC-MS	[51]
475	Atropine	C_17_H_23_NO_3_	289.1678	Fruit	HPLC-MS	[51]
476	Actinidine	C_10_H_13_N	147.1048	Fruit	HPLC-MS	[51]
477	5-Hydroxy-6-methoxy-7-O-*β*-D-glucopyranosyloxy-coumarin	C_16_H_18_O_10_	370.0900	Roots	HPLC-DAD-ESI-MS	[18]
478	Bis(2-ethylhexyl) phthalate	C_24_H_38_O_4_	390.2770	Roots	HPLC-DAD-ESI-MS	[18]
479	Argutosides E	C_24_H_24_O_12_	504.1268	Leaves	IR, ESI-TOF-MS, ^1^H-NMR, ^13^C-NMR, HMBC	[29]
480	Eculetin 7-O-(6′-O-*trans*-coumaroyl)-*β*-glucopyranoside	C_24_H_24_O_11_	488.1319	Leaves	IR, ESI-TOF-MS, ^1^H-NMR, ^13^C-NMR	[29]
481	Umbelliferone 7-O-(6′-O-*trans*-coumaroyl)-*β*-glucopyranoside	C_24_H_24_O_10_	472.1369	Leaves	IR, ESI-TOF-MS, ^1^H-NMR, ^13^C-NMR	[29]
482	Esculetin	C_9_H_6_O_4_	178.0266	Leaves	IR, ESI-TOF-MS, ^1^H-NMR, ^13^C-NMR	[29]
483	7,8-Dihydroxycoumarin	C_9_H_6_O_4_	178.0266	Leaves	IR, ESI-TOF-MS, ^1^H-NMR, ^13^C-NMR	[29]
484	Umbelliferone	C_9_H_6_O_3_	162.0317	Leaves	IR, ESI-TOF-MS, ^1^H-NMR, ^13^C-NMR	[29]
485	Aspartic acid	C_4_H_7_NO_4_	133.0375	Fruit, seeds	Amino acid analyzer	[53]
486	Threonine	C_4_H_9_NO_3_	119.0582	Fruit, seeds	Amino acid analyzer	[53]
487	Serine	C_3_H_7_NO_3_	105.0426	Fruit, seeds	Amino acid analyzer	[53]
488	Glutamate	C_5_H_9_NO_4_	147.0532	Fruit, seeds	Amino acid analyzer	[53]
489	Glycine	C_2_H_5_NO_2_	75.0320	Seeds	Amino acid analyzer	[53]
490	Alanine	C_3_H_7_NO_2_	89.0744	Seeds	Amino acid analyzer	[53]
491	Cystine	C_6_H_12_N_2_O_4_S_2_	240.0238	Fruit, seeds	Amino acid analyzer	[53]
492	Valine	C_5_H_11_NO_2_	117.0790	Fruit	Amino acid analyzer	[53]
493	Methionine	C_5_H_11_O_2_NS	149.0510	Fruit	Amino acid analyzer	[53]
494	isoleucine	C_6_H_13_NO_2_	131.0946	Fruit	Amino acid analyzer	[53]
495	Leucine	C_6_H_13_NO_2_	131.0946	Fruit	Amino acid analyzer	[53]
496	Tyrosine	C_9_H_11_NO_3_	181.0739	Fruit	Amino acid analyzer	[53]
497	Phenylalanine	C_9_H_11_NO_2_	165.0790	Fruit	Amino acid analyzer	[53]
498	Lysine	C_6_H_14_N_2_O_2_	146.1055	Fruit	Amino acid analyzer	[53]
499	Histidine	C_6_H_9_N_3_O_2_	155.0695	Fruit	Amino acid analyzer	[53]
500	Arginine	C_6_H_14_N_4_O_2_	147.1117	Fruit	Amino acid analyzer	[53]
501	Proline	C_5_H_9_NO_2_	115.0633	Seeds	Amino acid analyzer	[53]
502	Inositol	C_6_H_12_O_6_	180.0634	Roots	^1^H-NMR	[54]
503	Sucrose	C_12_H_22_O_11_	342.1162	Sprouts	HPLC	[39]
504	Glucose	C_6_H_12_O_6_	180.0634	Sprouts	HPLC	[39]
505	Fructose	C_6_H_12_O_6_	180.0634	Sprouts	HPLC	[39]
506	Maltose	C_12_H_22_O_11_	342.1162	Sprouts	HPLC	[39]
507	Xylose	C_5_H_10_O_5_	150.0528	Sprouts	HPLC	[39]
508	Emodin	C_15_H_10_O_5_	270.0528	Roots	HPLC	[52]
509	Chrysophanol	C_15_H_10_O_4_	254.0579	Roots	HPLC	[52]
510	3-Hydroxy-1-(4-O-*β*-D-glucopyranosyl-3-methoxyphenyl) propan-1-one	C_16_H_22_O_9_	358.1264	Fruit	ESI-MS, ^1^H-NMR, ^13^C-NMR	[21]

IR: infrared spectroscopy; TLC: thin-layer chromatography; HPLC-MS: high-performance liquid chromatography-mass spectrometry; ^13^C-NMR: carbon-13 nuclear magnetic resonance spectrometry; ^1^H-NMR: hydrogen-1 nuclear magnetic resonance spectrometry; HMBC: 1H-detected heteronuclear multiple bond correlation; ESI-MS: electrospray ionization-mass spectrometry; EI-MS: electron ionization-mass spectrometry; HPLC: high-performance liquid chromatography; HRESI-TOF-MS: high-resolution electrospray ionization-time of flight-mass spectrometry; HPLC-DAD-ESI-MS: high-performance liquid chromatography-diode array detection-electrospray ionization mass spectrometry.

**Table 8:** Inorganic elements isolated from *Actinidia arguta*.

No.	Name	Formula	Exact Theoretical Molecular Weight	Source	Characterization Method	Refs.
511	Calcium	Ca	39.9626	Fruit, leaves, branches, stems, roots, velamina, fibers	FPD, ICP, AAS, AFS	[55]
512	Kalium	K	38.9637	Fruit, leaves, branches, stems, roots, velamina, fibers	FPD, ICP, AAS, AFS	[55]
513	Magnesium	Mg	23.9850	Fruit, leaves, branches, stems, roots, velamina, fibers	FPD, ICP, AAS, AFS	[55]
514	Phosphorus	P	30.9738	Fruit, leaves, branches, stems, roots, velamina, fibers	FPD, ICP, AAS, AFS	[55]
515	Sodium	Na	22.9898	Fruit, leaves, branches, stems, roots, velamina, fibers	FPD, ICP, AAS, AFS	[55]
516	Aluminium	Al	26.9815	Fruit, leaves, branches, stems, roots, velamina, fibers	FPD, ICP, AAS, AFS	[55]
517	Ferrum	Fe	55.9349	Fruit, leaves, branches, stems, roots, velamina, fibers	FPD, ICP, AAS, AFS	[55]
518	Barium	Ba	137.9052	Fruit, leaves, branches, stems, roots, velamina, fibers	FPD, ICP, AAS, AFS	[55]
519	Strontium	Sr	87.9056	Fruit, leaves, branches, stems, roots, velamina, fibers	FPD, ICP, AAS, AFS	[55]
520	Manganese	Mn	54.9380	Fruit, leaves, branches, stems, roots, velamina, fibers	FPD, ICP, AAS, AFS	[55]
521	Lithium	Li	7.0160	Fruit, leaves, branches, stems, roots, velamina, fibers	FPD, ICP, AAS, AFS	[55]
522	Zinc	Zn	63.9291	Fruit, leaves, branches, stems, roots, velamina, fibers	FPD, ICP, AAS, AFS	[55]
523	Boron	B	11.0093	Fruit, leaves, branches, stems, roots, velamina, fibers	FPD, ICP, AAS, AFS	[55]
524	Cuprum	Cu	62.9296	Fruit, leaves, branches, stems, roots, velamina, fibers	FPD, ICP, AAS, AFS	[55]
525	Titanium	Ti	47.9479	Fruit, leaves, branches, stems, roots, velamina, fibers	FPD, ICP, AAS, AFS	[55]
526	Molybdenum	Mo	97.9054	Fruit, leaves, branches, stems, roots, velamina, fibers	FPD, ICP, AAS, AFS	[55]
527	Lead	Pb	207.9766	Fruit, leaves, branches, stems, roots, velamina, fibers	FPD, ICP, AAS, AFS	[55]
528	Chromium	Cr	51.9405	Fruit, leaves, branches, stems, roots, velamina, fibers	FPD, ICP, AAS, AFS	[55]
529	Nickel	Ni	57.9353	Fruit, leaves, branches, stems, roots, velamina, fibers	FPD, ICP, AAS, AFS	[55]
530	Indium	In	114.9039	Fruit, leaves, branches, stems, roots, velamina, fibers	FPD, ICP, AAS, AFS	[55]
531	Vanadium	V	50.9440	Fruit, leaves, branches, stems, roots, velamina, fibers	FPD, ICP, AAS, AFS	[55]
532	Arsenic	As	74.9216	Fruit, leaves, branches, stems, roots, velamina, fibers	FPD, ICP, AAS, AFS	[55]
533	Zirconium	Zr	89.9047	Fruit, leaves, branches, stems, roots, velamina, fibers	FPD, ICP, AAS, AFS	[55]
534	Cobalt	Co	58.9332	Fruit, leaves, branches, stems, roots, velamina, fibers	FPD, ICP, AAS, AFS	[55]
535	Cadmium	Cd	113.9034	Fruit, leaves, branches, stems, roots, velamina, fibers	FPD, ICP, AAS, AFS	[55]
536	Mercury	Hg	201.9706	Fruit, leaves, branches, stems, roots, velamina, fibers	FPD, ICP, AAS, AFS	[55]
537	Beryllium	Be	9.0122	Fruit, leaves, branches, stems, roots, velamina, fibers	FPD, ICP, AAS, AFS	[55]
538	Selenium	Se	79.9165	Fruit, leaves, branches, stems, roots, velamina, fibers	FPD, ICP, AAS, AFS	[55]
539	Yttrium	Y	88.9058	Fruit, leaves, branches, stems, roots, velamina, fibers	FPD, ICP, AAS, AFS	[55]

FPD: flame photometric detector; AAS: atomic absorption spectroscopy; ICP: inductively coupled plasma; AFS: atomic fluorescence spectrometry.

## Data Availability

All data presented in this study are available in the article.
